# Enhancing medical students` confidence and performance in integrated structured clinical examinations (ISCE) through a novel near-peer, mixed model approach during the COVID-19 pandemic

**DOI:** 10.1186/s12909-022-03970-y

**Published:** 2023-02-23

**Authors:** Ravanth Baskaran, Srinjay Mukhopadhyay, Sashiananthan Ganesananthan, Movin Peramuna Gamage, Nishaanth Dalavaye, Vincent Ng, Richard Bennett, Sripradha Srinivasan, Parvathy Sureshkumarnair, Robert Spencer, Harsh Bhatt, Susruta Manivannan, Malik Zaben

**Affiliations:** 1 School of Medicine, Cardiff University, Heath Park Campus, Cardiff, CF14 4YS UK; 2 Chelsea and Westminister Hospital NHS Foundation Trust, 369 Fulham Rd, London, SW10 9NH UK; 3 Neuroscience and Mental Health Research Institute (NMHRI), Cardiff University, Cardiff, UK; 4 Department of Neurosurgery, University Hospital of Wales, Cardiff, UK; 5 Department of Neurosurgery, Southampton General Hospital, Tremona Road, Southampton, SO16 6YD UK

**Keywords:** Near-peer; medical education; clinical; integrated/objective structured clinical examination (ISCE/OSCE), Near-peer, Mixed modal, Medical education, Clinical assessment, OSCEs

## Abstract

**Background:**

Near-peer medical education serves as an important method of delivering education to junior students by senior students. Due to the reduced clinical exposure because of the COVID-19 pandemic, we developed a mentorship scheme to help medical students with their Integrated Structured Clinical Examinations (ISCEs) by providing a combination of near-peer mentorship together with lecture-based teaching on a weekly basis for a 12-week period. Students attended a specialty-focused lecture every Tuesday followed by a small group teaching session organised by their tutor.

**Methods:**

A longitudinal evaluative interventional study was undertaken by the international student led medical education organisation, OSCEazy. The teaching programme was organised and conducted by third year medical students to a recruited cohort of second year medical students. Students’ perceptions of ISCEs (confidence, anxiety, and overall performance) were evaluated using 5-point Likert scales while their knowledge of the specialty was assessed using 10 single best answer questions which were distributed via Google® forms at the start and end of each week. In addition, we assessed tutor perceptions of their teaching and learning experience.

**Results:**

Seventy-two tutees were enrolled in the programme (mean age: 24.4, female: 77.8%). 88.9% of the participants had not attended any online ISCE teaching prior to this. They preferred in-person ISCE teaching as compared to virtual sessions [median 4.5 (IQR 4–5) vs 3 (IQR 3–4), *p* <  0.0001), respectively]. There was a significant overall increase in knowledge when comparing pre-session and post-session performance [mean 53.7% vs 70.7%, *p* <  0.0001)]. There was a significant increase in student confidence [Confidence: median 3 (IQR:3–4) vs 4 (IQR 3–4), *p* <  0.0001] while no change was seen in the anxiety and perception of their overall performance in an ISCE. [Anxiety: median 3 (IQR 2–4) vs 3 (IQR 3–4), *p* = 0.37, Performance: median 3 (IQR 3–4) vs median 3 (IQR 3–4), *p* <  0.0001]. The tutors reported an increase in their confidence in teaching ISCEs online [median 3 (IQR 2–3.25) vs median 4 (IQR 4–5), *p* <  0.0001)].

**Conclusion:**

Online near-peer teaching increases the confidence of both tutees and tutors involved while enhancing the tutees’ knowledge of the specialty. Thus, medical schools should incorporate near-peer teaching in their curriculum to enhance the student learning experience.

**Supplementary Information:**

The online version contains supplementary material available at 10.1186/s12909-022-03970-y.

## Background

The COVID-19 pandemic has made a huge impact on the delivery of medical education. To teach clinical medicine, medical school curricula often allocate medical students to hospitals to provide clinical exposure [1]. However, due to the risk of contracting, spreading, and facing the impacts of COVID-19, medical schools in the United Kingdom have had to discontinue or cancel clinical placements for a sustained period [2]. To combat this, many students lend themselves to other forms of learning such as online or virtual learning platforms. However, medicine invariably requires elements of practical learning, not only for essential procedural skills, but also to develop consultation skills such as the art of eliciting a comprehensive history [2]. The latter is typically gained through repetition throughout medical school by spending time shadowing clinicians and by regular patient-student interactions [3, 4] . However, the COVID-19 pandemic has forced medical schools and student organisations to employ online teaching methods to deliver medical education on a consistent basis in an attempt to replace clinical placements temporarily.

Prior to the COVID-19 pandemic, there has been a significant change in the delivery of medical education, from a model of didactic, lecture-based teaching, to one of problem-based, facilitated small group teaching [5, 6]. This is due to the recognition that traditional didactic techniques limit student interaction and often present too large a volume of information for students to reasonably be able to understand and retain [7, 8]. In contrast, small group methods foster camaraderie among participants and group learning [9, 10]. Nevertheless, the logistical requirements for organising multiple small groups are significant when compared to that of a single lecturer giving a talk to a single cohort. The COVID-19 pandemic has brought virtual platforms such as Zoom™ to the fore, meaning that didactic knowledge can be shared without geographical constraints. Furthermore, a recent systematic review has shown that there is no clear evidence to suggest that offline (in-person) learning is superior to online learning [10, 11].

Near-peer education is a technique that has gained popularity in recent years. Studies have demonstrated the value of teaching delivered by individuals who are only slightly more senior than the learners (so-called ‘near-peers’), demonstrating positive outcomes in certain contexts, including that of clinical examinations [12–15]. Evidence further suggests that near-peer teachers can frame their teaching methods around their personal level of understanding of concepts, which fosters a more relatable and positive learning environment. This allows students to connect on a more personal level compared to faculty members [16–19]. Additionally, near-peer teaching also benefits teachers. Despite the increasing expectations for medical graduates to teach and lead, training and guidance of teaching skills are not commonly provided [18]. Near-peer teaching allows for tutors to improve their teaching skills, consolidate and recall past knowledge and develop their confidence as leaders and educators [20, 21].

Near-peer teaching is useful in all forms of medical education. However, it is shown to be particularly helpful in Integrated Structured Clinical Examination (ISCE). The ISCE is a modified version of the commonly known Objective Structured Clinical Examination (OSCE). Similar to the OSCEs, it assesses the history taking, communication, clinical skills, procedural skills and data interpretation aspects of medicine. Each aforementioned component of the ISCE is integrated to simulate holistic care for patients. For example, a single ISCE station may involve history-taking for meningitis, practical administration of intramuscular benzylpenicillin, and diagnostic interpretation of lumbar puncture results. ISCE examinations are typically performed at critical transition points throughout medical school.

OSCEazy is an international organisation that was set up in the midst of the COVID-19 pandemic (March 2021) in order to cater to lack of ISCE/OSCE in an online format. It has since expanded to teach clinical, surgical, research, preclinical, premedical and post graduate content to the masses. Together with the online lectures that were organised, the local chapter of OSCEazy arranges a near-peer ISCE teaching programme for students in Cardiff University School of Medicine. This allows tutees to gain valuable skills and tips from seniors who have recently sat the examination.

Through our programme, we aimed to integrate virtual learning, near-peer teaching, lecture-based teaching, and small group teaching to provide an effective 4-week course for second year medical students. Herein, we report the results of our mixed-modality teaching course from the perspective of tutors and tutees, and its effects on clinical confidence and performance in the end-of-year Integrated Structured Clinical Examination (ISCE).

## Methods

### Participant recruitment

This longitudinal evaluative interventional study was performed via a local chapter (Cardiff University) of the international medical student led organisation, OSCEazy. Study participants were second-year medical students, and all teaching sessions were delivered by third-year medical students. Tutees and tutors were selected by invitations disseminated via email and group messages to the entire cohort of second- and third-year medical students respectively. All year 2 medical students who applied were taken into the programme and the appropriate number of tutors were recruited thereafter to have a tutor to tutee ratio of 1:2 on a first-come first serve basis.

### Intervention

**Fig. 1:**
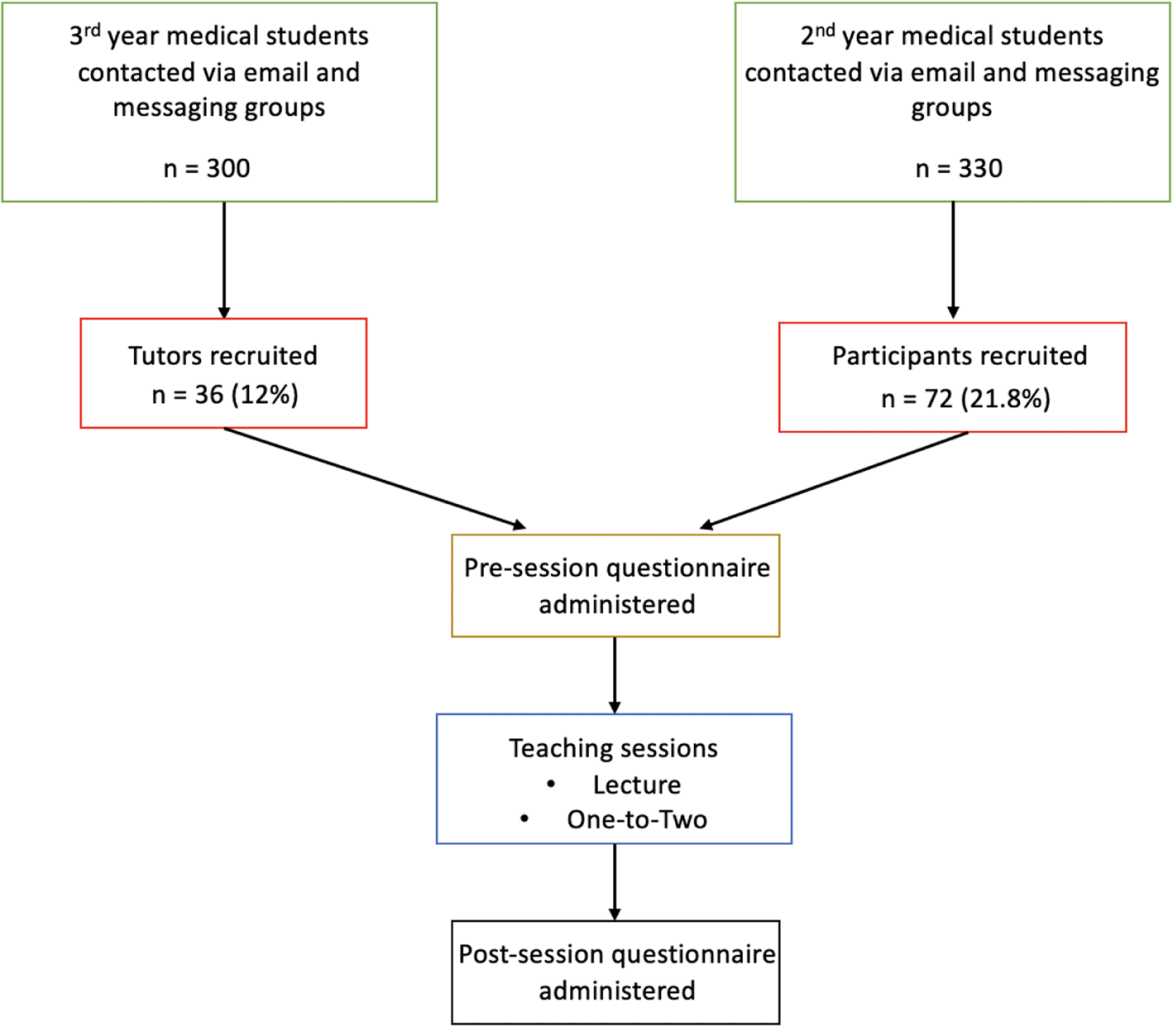
Flowchart of chronological event structure

Lecture style teaching sessions were delivered once-weekly (every Tuesday) over a 12-week period on the following medical specialties: Cardiology, Respiratory Medicine, Gastroenterology, and Neurology. Each virtual session was delivered across the online platform Zoom™ and comprised of a one-hour virtual lecture followed by a small group session. The small group session (‘one-to-two programme’) involved one third-year student and 2 second-year students, covering a simulated ISCE scenario relating to the preceding lecture (Fig. 1). In order to assess the effects of the intervention, we obtained responses to surveys circulated prior to (pre-session questionnaire) and following (post-session questionnaire) the sessions on a weekly basis for 4 weeks, evaluating two domains: (i) the change in students’ perceptions of the ISCE format (confidence, examination anxiety and perception of personal performance); and (ii) the change in performance on knowledge-based questions relevant to that week’s specialty.

**Fig. 2:**
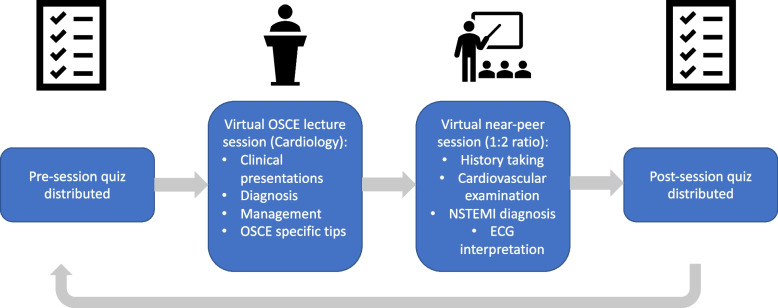
Schematic of example teaching intervention workflow. Different specialties were covered over a 12-week period

*Example run-through of a programme week (*Fig. 2***).***

*Specific details of each session and material from the cardiology session are outlined in* *Appendix* 1.

### Questionnaire design and dissemination

Questionnaires were distributed as described above using anonymised Google® Forms. Perceptions of ISCE assessment were evaluated using five-point Likert scales. Clinical knowledge was assessed using ten standardised single best answer questions with 5 options, written by lecturers and peer-reviewed by a minimum of 3 other tutors. Sample questions from one pre-session and post session questionnaire have been detailed in the Appendix.

### Ethical considerations

This study constituted an evaluation of a voluntary novel teaching format. Approval was sought from the Cardiff University School of Medicine Research Ethics Committee who confirmed that formal approval was not required. Questionnaires contained relevant information about the study and stated that completion of the survey assumed consent for use of anonymised data in future presentation and publication.

### Statistical analysis

Summary statistics were reported as appropriate for baseline characteristics. Shapiro-Wilk test was used to test for the normality of data distribution, where a threshold of > 0.05 was considered to indicate normal distribution. Where more than one questionnaire was distributed for pre and post evaluations, the non-parametric unpaired Wilcoxon rank sum test with continuity correction was utilised (questions for tutees across the programme) however, when this was based on one questionnaire (tutors’ questionnaire), a paired analysis was utilised of the same test.

Binary proportion comparisons of pre and post knowledge scores was determined using a McNemar’s Chi-squared test. For all statistical analyses, a *p*-value threshold of < 0.05 was considered to indicate statistical significance. All statistical analyses were performed in R.

## Results

### Study population

Seventy-two out of 330 (21.8%) second-year students were enrolled on the one-to-two programme. The mean age of the tutees was 24.4 years, with 77.8% being female. 89% of the tutees stated they had not attended any form of virtual ISCE/OSCE teaching prior to the present programme.

#### Questionnaire analysis: tutee responses

Analysis of the initial tutee perceptions showed that they felt more confident being taught ISCE content in-person as opposed to in a virtual format [median 4.5 (IQR:4–5) vs 3 (IQR:3–4) respectively, *p* <  0.0001)] (Table 1).

**Table 1:** Table showing the **perception** of confidence of tutees in receiving ISCE teaching virtually and in-person

	Median (IQR)	***p***-value
Confidence of tutees in receiving ISCE teaching through a **virtual** format	3.0 (3–4)	< 0.0001
Confidence of tutees in receiving ISCE teaching **in-person**	4.5 (4–5)	

Tutee performance in knowledge-based questions improved significantly with the teaching intervention. In analysing the percentage of correct answers in the pre-session questionnaire compared to the post-session questionnaire, there was a significant increase in the mean percentage correct (mean difference 17.0%, *p* <  0.001) (Table 2).

**Table 2:** Table showing the mean value of correct responses to the knowledge assessments completed before and after each teaching session

	Correct answers (mean)	***p***-value
Knowledge assessment in pre-questionnaires	53.7%	< 0.0001
Knowledge assessment in post-questionnaires	70.7%	

The confidence of tutees with respect to sitting their upcoming ISCE improved over the course of the programme. However, despite this improvement in confidence, there was no significant change in the anxiety tutees felt towards the ISCE (pre-median 3 (IQR:2–4) vs post-median 3 (IQR:3–4), *p* = 0.37). Similarly, there was no change in the tutees’ perception of how well they will do (overall performance) in their ISCE over the course of the programme (Table 3).

**Table 3:** Table showing the confidence, anxiety, and self-assessed perception of ISCE performance of tutees at the start and end of the teaching programme

	Pre-Questionnaire (median (IQR))	Post- Questionnaire (median (IQR))	p-value
Confidence of tutees in performing well in their ISCE before and after the programme	3.0 (3–4)	4.0 (3–4)	< 0.0001
Anxiety of tutees towards their ISCE before and after the programme	3.0 (2–4)	3.0 (3–4)	0.37
Perception of tutees’ overall performance in their ISCE before and after the programme (self-assessed)	3.0 (3–4)	3.0 (3–4)	< 0.0001

#### Questionnaire analysis: tutor responses

Among the 36 out of 300 (12%) third-year tutors who volunteered to delivered the teaching programme, an increase in confidence in delivering ISCE teaching virtually over the course of the programme was evident [median 3 (IQR:2–3.25) vs 4 (IQR:4–5), *p* <  0.0001)]. Similarly, tutors reported an increase in their confidence in delivering such teaching in-person [median 3 (IQR:2–4) vs 4 (IQR:3–4), *p* < 0.0001)] (Table 4).

**Table 4:** Table showing the tutor **perceptions** towards the delivery of ISCE teaching

	Pre- Questionnaire (median (IQR))	Post- Questionnaire (median (IQR))	***p***-value
Confidence of tutors in delivering ISCE teaching through a virtual format at the start and end the programme	3.0 (2–3.25)	4.0 (3–5)	< 0.0001
Confidence of tutors in delivering ISCE teaching in-person at the start and end of the programme	3.0 (2–4)	4.0 (3–4)	< 0.0001

## Discussion

The COVID-19 pandemic has represented an unprecedented challenge to medical educators and medical students alike. The shift to online/distance learning during the early phases of the pandemic meant that there was a significant loss of face-to-face clinical learning opportunities for early years medical students. We identified an opportunity to deliver a structured, weekly virtual teaching programme aimed specifically at improving students’ preparedness for the examination utilising a near-peer learning model. To our knowledge, this is the first near-peer mixed-modality clinical teaching programme described and evaluated. We have shown that this model is effective not only in improving students’ knowledge (Table 2), but also improving their confidence going into the real-world examination (Table 3). Moreover, highly positive feedback was received from the third year near-peer tutors, identifying significant improvement in their confidence in delivering such teaching (Table 4) [21].

Near-peer teaching is not new to medical education. This teaching model leverages the principles of cognitive and social congruence between the student and near-peer teacher. Cognitive congruence refers to the similar knowledge base shared by near-peer tutors with their tutees, resulting in tutors understanding the likely gaps in their knowledge/skills and being able to deliver the content at an appropriate level [9, 17]. By comparison, social congruence occurs due to the common or similar social role shared by tutor and tutee [7]. The social and cognitive congruence between near-peer tutors and tutees fosters a pleasant, serene and motivational work environment [5, 12, 18]. It allows for supportive learning with ease in understanding concepts, tutees feeling able to ask for clarification when required, as well as providing constructive feedback to the tutor [22]. Near-peer education has been shown to improve students’ motivation for study, problem-solving, organisational and time management skills [11]. These skills are said to be less well addressed by senior-led teaching as faculty are separated from tutees in terms of both experience and relatability. Conversely, near-peer-educators recall their examination experiences and guide their tutees with respect to the specifics of the examination and the preparation required. The near-peer tutors, based on personal experiences, are therefore able to segregate essential and trivial information, hence imparting specific knowledge to their tutees [9, 14]. This concept is underpinned by work by Ten Cate et al. and Whitman et al. who suggest that peer-educators have a ‘conscious competence’ of a recently completed examination as compared to senior faculty who possess an ‘unconscious competence’, resulting in them being less skilled in providing highly specific advice and conveying concepts which novices struggle to understand [14, 15, 19].

Interestingly, our results indicate that the overall academic performance and confidence of the tutees increased throughout the programme while there was no significant change observed in their anxiety and the perception of their performance in an ISCE. It has been shown previously that confidence and anxiety are unrelated to a student’s eventual examination performance [4]. However, others have shown that in the context of clinical examinations, practice in the form of formative assessments improves examination outcomes [5]. Anxiety is conventionally divided into ‘trait’ and ‘state’ forms. State anxiety is defined as the temporary reaction to an adverse event while trait anxiety is a more perpetual prolonged form of anxiety associated with one’s personality. We could hypothesise that students in our study are experiencing trait anxiety due to the continuous barrage of medical education provided to them coupled with the overbearing constant worry of approaching ISCEs and other examinations [6].

The Yerkes-Dodson law suggests that a small degree of state anxiety enhances performance. However, greater anxiety/stress levels would be more likely to adversely affect student performance in an ISCE [23]. The participants in this study demonstrated an improvement in knowledge as well as confidence. Therefore, their anxiety appears to be of a ‘trait’ type given the unchanged nature of anxiety levels despite objective improvement in preparedness. This might be a result of the continuous academic pressures associated with medical school, the wider societal uncertainties associated with the pandemic, or indeed an amalgamation of factors.

### Tutors’ perceptions

Medicine in general relies on a hierarchical transfer of knowledge [9, 14, 17, 19]. This consistent transfer requires competent professionals that are willing and have the skills, capacity, and motivation to teach their juniors. Our study adds to the body of literature suggesting that tutors who have just experienced examinations are the most valuable for junior students, in certain contexts. This value arises from relatability as well as a first-hand experience of the upcoming examination providing ease of communication and cognitive congruence [24].

In order to successfully teach a topic, a tutor must have sufficient depth of understanding for the intended session. Tutors therefore need to prepare adequately to be able to support juniors through their learning journey [25, 26]. This involves in-depth content preparation and planning of delivery method in order to convey the information in an understandable manner, as well as being able to answer any questions posed by the tutee [12].

Our cohort of tutors found that their confidence in teaching ISCEs online improved through the duration of the course. Furthermore, they felt that their confidence in teaching ISCEs in-person increased because of participating in this virtual programme. Sawyer et al. suggest that teaching improves grades in the tutors’ own coursework [27–29]. Such analyses are beyond the scope of the present study, but Whitman et al. similarly suggest that ‘to teach is to learn twice’ which links with our postulation that tutors gain an academic advantage through the process of imparting knowledge to their juniors [13]. Thus, our near-peer teaching programme is of benefit not only to the tutees, but to the tutors as well.

Our findings are largely consistent with recently published studies in the literature [30]. One single centre study based in the USA similarly demonstrated mutual benefits to fourth- and second-year medical students in a near-peer teaching paradigm [19]. In contrast to our study, however, tutors were provided with orientation workshops. Furthermore, this allowed fourth year medical students to develop their skills as educators [19]. Another single centre study based in the USA built upon this conclusion with a one-year longitudinal study assessing near-peer teaching [31]. The authors highlighted that near-peer teaching reiterates pedagogy in medical education and prepares medical students to serve as educators in their careers as clinicians [31]. More recently, a single centre study based in Switzerland demonstrated the value of near-peer feedback in formative online OSCE sessions [32]. Participants highlighted the empathy and relevance of feedback from their peers [32]. Simultaneously, we see in the study conducted by Grover et al. that students found virtual OSCE sessions teaching improving their clinical skills and found it as interactive and engaging compared to in-person OSCE sessions [33]. This contrasts with our findings, which suggested that students did not consider in-person teaching to be entirely replaceable by virtual sessions. With increasing demands on healthcare professionals, we see that to accommodate and receive high quality medical education, near-peer teaching plays a crucial role. We believe that organisations and institutions should try to integrate near-peer teaching into their curriculum such that it becomes a part of the medical school experience.

### Strengths and limitations

This study showed that near-peer medical education is a format of teaching that can be utilised by organisations and medical schools in delivering effective teaching. By recruiting senior medical students to tutor junior medical students, it not only reduces the burden on educational institutions to provide supplementary teaching but also allows for students to feel well supported. It confers educational and leadership skills to senior medical students that would make them better leaders and educators, a requirement for medical graduates as they progress through the ranks.

This study has several limitations. Firstly, our analysis method of using bi-weekly surveys targeting similar domains may have induced an element of ‘feedback fatigue’ among participants, potentially skewing responses in some way [8]. There is also potential for heterogeneity in the experience of tutees depending on which tutor was facilitating their one-to-two sessions. This might relate to differing personalities, teaching styles and/or delivery of content by individual tutors that is beyond our control. This was a single-centre study with a relatively small cohort (tutees: *n* = 72; tutors: *n* = 36). Hence, data might not be able to be generalised. Moreover, 77.8% of our tutee cohort were female, thus the levels of anxiety seen in our study might be different in a more balanced group, given known gender-specific differences in levels of test anxiety [34]. Additionally, this study does not link the actual performance of a second-year medical student to the ISCEs. In order to completely assess this, a study that ties in this course to the examination results of the cohort would have to be undertaken.

## Conclusion

Delivering education online remains a novel and convenient method of disseminating knowledge to large cohorts, especially in the context of the COVID-19 pandemic. In our study, we demonstrate that near-peer, multi-modality teaching has increased knowledge among tutees and confidence among both tutors and tutees. In future, medical education should adopt a multi-modal approach which leverages the positives of both in-person and virtual clinical teaching. The integration of such virtual interactions into the medical curricula would allow for students to be well equipped for the future where elements of patient care are likely to move onto contactless platforms [35]. 

## Supplementary Information

**Additional file 1.**

**Additional file 2.**

**Additional file 3.**

**Additional file 4.**

## Data Availability

The datasets generated and/or analysed during the current study are available in the ‘Raw Data’ repository, in Supplementary Materials. All data was collected and stored according to General Data Protection Regulation (GDPR) guidelines. All methods were carried out in accordance with the relevant guidelines and regulations. All participants were made aware and informed of the study. All participants provided informed consent.
