# Supplementary material for: Enhancing medical students` confidence and performance in integrated structured clinical examinations (ISCE) through a novel near-peer, mixed model approach during the COVID-19 pandemic
Source: BMC Med Educ. 2023 Feb 23;23:128. doi: 10.1186/s12909-022-03970-y (PMC9947444; doi:10.1186/s12909-022-03970-y)
Supplement: Supplementary file 1 — Additional file 1. [file 12909_2022_3970_MOESM1_ESM.docx]

**Appendix**

Sample questions (taken from respiratory case)

*Pre-cardiology Quiz*

Each quiz is started by common questions followed by case-based SBAs.

| **Gender?** |
| --- |
| Male |
| Female |
| Other |
| Prefer not to Say |
| **Age?** |
| (Text box) |
| **Have you attended any previous ONLINE ISCE/OSCE teaching?** |
| Yes |
| No |
| **How confident do you feel being taught ISCE (quality of teaching) IN-PERSON.** |
| 1 (Not very confident) – 5 (Very confident) |
| **To what extent are you worried that an ONLINE format of teaching ISCE may affect your overall performance in a real ISCE examination.** |
| 1(Not worried at all) – 5(Extremely worried) |
| **To what extent are you worried that an ONLINE format of teaching ISCE may affect your overall performance in a real ISCE examination.** |
| Asking questions |
| Listening clearly |
| Speaking in parallel |
| Surrounding factors (noise, other household members) |
| Quality of Internet |
| Unreliability of my device |
| Difficulty being rapport with tutor |
| *Other* (Text box) |
| **If you DO NOT do well in an ISCE, to what extent do you think ONLINE teaching of ISCE could be a contributor?** |
| 1 (Irrelevant factor) – 5 (Very strong factor) |
| **If you do well in an ISCE, to what extent do you think ONLINE teaching of ISCE could be a contributor?** |
| 1 (Irrelevant factor) – 5 (Very strong factor) |
| **To what extent would an ONLINE format of ISCE teaching affect your ANXIETY in excelling in real ISCEs** |
| 1 (Much less anxious) – 5 (Much more anxious) |
| **To what extent will an ONLINE format of ISCE teaching affect my OVERALL PERFORMANCE in a real ISCE?** |
| 1 (Much more negatively) – 5 (Much more positively) |
| **Examination: You are on a cardiology ward examining a patient. While doing a manoeuvre, you notice that there is a change in his radial pulse. You identify the condition as aortic regurgitation. Which of the following signs is most likely associated with aortic regurgitation?** |
| Bounding pulse |
| Oscillating pulse |
| Slow-rising pulse |
| Vibrating pulse |
| Water-hammer pulse |
| **Data Interpretation: A 60 y/o male patient presents with intermittent dizziness, reports one episode of syncope and upon examination, his pulse is 45 bpm. A diagnosis is suspected and an ECG is done. What is the ECG showing?** |

| 1st degree heart block |
| --- |
| 2nd degree mobitz type 1 heart block |
| 2nd degree mobitz type 2 heart block |
| 3rd degree heart block |
| Left bundle branch block |
| **Examination: At which location would you auscultate a pansystolic murmur that is associated with pulmonary blood flow?** |
| Black |
| Green |
| Orange |
| Red |
| Yellow |
| **Management: A 58 y/o man arrives at A&E complaining of a 3 hour history of tight chest pain radiating to the jaw and left arm. The ECG confirms NSTEMI and blood tests show an elevated troponin. The patient has an oxygen saturation of 94% on air. What is the most appropriate initial management out of the following options?** |
| Beta-blocker |
| GTN |
| Oxygen |
| PCI |
| Thrombolysis |
| **HISTORY: You have been asked by the GP to take a history from a 67 year old male who is complaining of chest pain. Which of the following from the history would point towards a diagnosis of stable angina?** |
| Burning sensation in the chest |
| Central crushing chest pain during exertion |
| Pain which is worse on inspiration |
| Sharp, stabbing chest pain which is worse when lying down |
| Sharp, tearing pain radiating to the back |
| **Clinical Skill: A 58 y/o female patient comes into A&E complaining of central, crushing chest pain and the attending physician decides to conduct an ECG. In terms of chest lead placement, which lead is placed at the 5th intercostal space, left midclavicular line?** |
| V1 |
| V2 |
| V3 |
| V4 |
| V5 |
| **Clinical Skill: A 2nd year medical student is waiting for their bus at the bus stop when he notices a nearby man collapse to the ground. As someone who has recently done basic adult life support training, he decides to assess the surrounding for any imminent danger to either him or the man. In terms of assessment and resuscitation, what is next best step?** |
| Assess patient for response |
| Inspect airway + assess breathing |
| Shout for help |
| Start chest compressions |
| Use defibrillator |
| **Differential: A patient presents to the A&E with sharp, stabbing chest pain, which is worse when he lies down, but is better when he sits forward. He has a myocardial infarction, 4 days ago and was sent home after having stents placed in his LAD and LCx. What is the most like diagnosis?** |
| Cardiac Tamponade |
| Dressler's syndrome |
| Left ventricular free wall rupture |
| Pericarditis |
| Ventricular septal defect |
| **Data Interpretation: A 70 year old man presents with severe, central, crushing chest pain. His ECG trace is shown below. What does it show?** |
| Anterior STEMI |
| Atrial Fibrillation |
| Anterolateral STEMI |
| Left Bundle Branch Block |
| Posterior MI |
| **History: A 62 y/o female patient presents to the hospital with symptoms of left sided heart failure. Which of the following is NOT a symptom of left sided heart failure?** |
| Exertional dyspnoea |
| Fatigue |
| Paroxysmal nocturnal dyspnoea |
| Productive cough |
| Swollen legs |

*Post-cardiology quiz*

Each quiz is started by common questions followed by case-based SBAs.

| **Gender?** |
| --- |
| Male |
| Female |
| Other |
| Prefer not to Say |
| **Age?** |
| (Text box) |
| **How confident do you feel being taught ISCE (quality of teaching) using an ONLINE format?** |
| 1 (Not very confident) – 5 (Very confident) |
| **How confident do you feel being taught ISCE (quality of teaching) IN-PERSON?** |
| 1 (Not very confident) – 5 (Very confident) |
| **To what extent are you worried that an ONLINE format of teaching ISCE may affect your overall performance in a real ISCE examination.** |
| 1 (Not worried at all) – 5 (Extremely worried) |
| **What factors are you worried about being taught ISCE online (lecture and tutorial)?** |
| Asking questions |
| Listening clearly |
| Speaking in parallel |
| Surrounding factors (noise, other household members) |
| Quality of Internet |
| Unreliability of my device |
| Difficulty being rapport with tutor |
| (Textbox for others) |
| **If you DO NOT do well in an ISCE, to what extent do you think ONLINE teaching of ISCE could be a contributor?** |
| 1 (Irrelevant factor) – 5 (Very strong factor) |
| **If you DO well in the real ISCE, to what extent do you think ONLINE teaching of ISCE could be a contributor?** |
| 1 (Irrelevant factor) – 5 (Very strong factor) |
| **To what extent would an ONLINE format of ISCE teaching affect your ANXIETY in excelling in real ISCEs** |
| 1 (Much less anxious) – 5 (Much more anxious) |
| **To what extent will an ONLINE format of ISCE teaching affect my OVERALL PERFORMANCE in a real ISCE?** |
| 1 (Much more negatively) – 5 (Much more positively) |
| **Data Interpretation: A 72-year-old woman presents to the emergency department with crushing, central chest pain, dyspnoea and excessive sweating. On admission, an ECG is taken. What is the most likely cause according to the ECG below?** |
| Anterolateral STEMI |
| Inferior STEMI |
| Lateral STEMI |
| NSTEMI |
| Pericarditis |
| **Data Interpretation: A 70 year old man presents with severe, central crushing pain. His ECG trace is shown below. What does it show?**  **PaO_2_: 11 kPa (11-13 kPa)** |
| Anterior STEMI |
| Atrial Fibrillation |
| Anterolateral STEMI |
| Left Bundle Branch Block |
| Inferior STEMI |
| **History: A 67 y/o male presents to A&E acutely with chest pain. Which of the following features in the patient's history makes it most likely to be aortic dissection?** |
| Chest pain that is bought on after eating |
| Chest pain that radiates to jaw or left arm |
| Chest pain that radiates to the back |
| Chest pain worse on inhalation |
| Chest pain worse on movement |
| **HISTORY: You are taking a history form a 86 year old male patient, complaining of palpitations which come on and stay for a few hours and then go away. Which of the following tests would you conduct next based on this history?** |
| Basic observations (Heart rate, temperature, respiratory rate etc), ECG, urine dip |
| CXR and Troponin levels |
| CT angiogram |
| Transthoracic Echocardiogram |
| Transoesophageal Echocardiogram |
| **Differential Diagnosis: A 40 year old female patient presents to A&E with dyspnoea and tachycardia. She was in a road traffic accident that resulted in blunt force trauma to the chest. On examination, she has muffled heart sounds, raised JVP and a BP of 90/68 mmHg. What condition is the patient most likely to have?** |
| Atrial fibrillation |
| Atrial flutter |
| Cardiac tamponade |
| Constrictive pericarditis |
| Infective endocarditis |
| **Examination: What is the condition that you would most associate narrowing of nail bed spacing when the two index fingers are put together with?** |
| Atrial fibrillation |
| First-degree heart block |
| Infective endocarditis |
| Pericarditis |
| Wolf-Parkinson White syndrome |
| **Examination: Greater than how many centimetres signifies a pathological Jugular Venous Pressure (JVP)?** |
| 2cm |
| 3cm |
| 4cm |
| 5cm |
| 6cm |
| **Management: A patient presents with severe central crushing chest pain. An ECG shows ST segment elevation in leads I, aVF, v5 and v6. What is the DEFINITIVE management of this condition?** |
| Pain relief |
| PCI |
| Oxygen therapy |
| Thrombolysis |
| Wait and Watch |
| **Clinical Skills: A 60 y/o male patient comes to cardiology clinic for a check up and the consultant decides to do an ECG. In terms of lead placement, where will the lead V2 (chest lead) be placed?** |
| 4th intercostal space, left sternal edge |
| 4th intercostal space, right sternal edge |
| 5th intercostal space, left midclavicular line |
| Left mid-axillary line |
| Medial or lateral malleolus of right leg |
| **Clinical Skills: A 50 y/o man goes into cardiac arrest in a ward and the attending nurse decides to perform basic life support on the patient. The nurse has reached the point of performing chest compressions and ventilating the patient. Which of the following must NOT be done when doing this?** |
| Observe the rise and fall of the patient’s chest when ventilating patient |
| Perform chest compressions and ventilation at 15:2 ratio |
| Pinch the nostrils when ventilating patient |
| Place a tight seal around patient’s mouth when ventilating patient |
| Place one hand over the other when performing chest compressions |

*Pre-respiratory Quiz*

Each quiz is started by common questions followed by case-based SBAs.

| **Gender?** |
| --- |
| Male |
| Female |
| Other |
| Prefer not to Say |
| **Age?** |
| (Text box) |
| **How confident do you feel being taught ISCE (quality of teaching) using an ONLINE format?** |
| 1 (Not very confident) – 5 (Very confident) |
| **To what extent are you worried that an ONLINE format of teaching ISCE may affect your overall performance in a real ISCE examination.** |
| 1 (Not worried at all) – 5 (Extremely worried) |
| **What factors are you worried about being taught ISCE online (lecture and tutorial)?** |
| Asking questions |
| Listening clearly |
| Speaking in parallel |
| Surrounding factors (noise, other household members) |
| Quality of Internet |
| Unreliability of my device |
| Difficulty being rapport with tutor |
| (Textbox for others) |
| **If you DO NOT do well in an ISCE, to what extent do you think ONLINE teaching of ISCE could be a contributor?** |
| 1 (Irrelevant factor) – 5 (Very strong factor) |
| **If you DO well in the real ISCE, to what extent do you think ONLINE teaching of ISCE could be a contributor?** |
| 1 (Irrelevant factor) – 5 (Very strong factor) |
| **To what extent would an ONLINE format of ISCE teaching affect your ANXIETY in excelling in real ISCEs** |
| 1 (Much less anxious) – 5 (Much more anxious) |
| **To what extent will an ONLINE format of ISCE teaching affect my OVERALL PERFORMANCE in a real ISCE?** |
| 1 (Much more negatively) – 5 (Much more positively) |
| **History: A 24 year old cystic fibrosis patient presents to the respiratory clinic. He mentions a series of symptoms which drive the respiratory consultant to make a diagnosis of Bronchiectasis. Which of the following is most likely to be associated with bronchiectasis?** |
| Coughing up pink, frothy sputum |
| Coughing up cup full of green, purulent sputum |
| Dry cough, worse at night |
| Haemoptysis |
| Severe shortness of breath on exertion |

| **Examination: You are conducting a respiratory examination on a patient with long-term COPD when you notice asterixis. What is the most likely cause of this in this patient?** |
| --- |
| Beta-2 agonist use |
| Carbon dioxide retention |
| Hepatic encephalopathy |
| Thyrotoxicosis |
| Uraemia |
| **Data Interpretation: What is the seen on this CT image?**  **A Picture of CT scan showing Honey Combing in Idiopathic Pulmonary Fibrosis*** |
| Emphysema |
| Honey combing |
| Pleural Plaques |
| Singlet rings |
| Tram lines |
| **Data Interpretation: A patient with a know history of COPD presents to hospital with shortness of breath, cough and wheeze. An ABG is done and the results are shown below. What is the ABG showing?**  **PaO_2_: 9.5 kPa (11-13 kPa)**  **pH: 7.14 (7.35 – 7.45)**  **PaCO_2_: 8.1 kPa (4.7 – 6.0 kPa)**  **HCO_3_^-^: 15.2 (22 – 26 mEq/L)**  **BE: -9.7 (-2 to +2)** |
| Metabolic acidosis |
| Metabolic acidosis with respiratory compensation |
| Mixed respiratory and metabolic acidosis |
| Respiratory acidosis |
| Respiratory acidosis with metabolic compensation |
| **Clinical Skill: You are placed in a GP in North Wales, where a patient walks in complaining of a nocturnal cough, and chest tightness. The GP asks you to explain to the patient how to conduct a Peak Flow Assessment. Which of the following is essential to mention to the patient in order to get accurate readings.** |
| Breathe in through the meter and then breathe out |
| Breathe out normally through the peak flow meter |
| Lie flat during the assessment |
| Repeat the Process at least 5 times and take the average |
| Stand up during the assessment |
| **Clinical Skill: You are a medical student shadowing a GP at their practice when you are asked by the GP to explain how to correctly use an inhaler. The student explains how to use an inhaler to the best of their knowledge. Out of the steps mentioned below, which is INCORRECT?** |
| Check expiry date |
| Cover the mouthpiece using a tight seal |
| Depress the inhaler cannister before you start breathing in |
| Sit upright before breathing in |
| Wait up to 1 minute before repeating |
| **Differential: A 48 year old lady presents to the GP complaining of sudden onset severe left sided chest pain, which is worse on inspiration. She is currently being treated for active breast cancer. She is a non-smoker, but does have a BMI of 36 kg/m2. Based on the above description what is the most likely diagnosis?** |
| Asthma |
| Bronchiectasis |
| COPD |
| Pneumothorax |
| Pulmonary embolism |
| **Management: A 42 y/o female patient comes into hospital with pyrexia, productive cough & dyspnoea. A diagnosis of community-acquired pneumonia is made. The patient states that she is allergic to penicillin. Given this information, what is the most appropriate antibiotic to be prescribed?** |
| Amoxicillin |
| Co-amoxiclav |
| Doxycycline |
| Gentamicin |
| Metronidazole |

*Post-respiratory quiz*

Each quiz is started by common questions followed by case-based SBAs.

| **Gender?** |
| --- |
| Male |
| Female |
| Other |
| Prefer not to Say |
| **Age?** |
| (Text box) |
| **How confident do you feel being taught ISCE (quality of teaching) using an ONLINE format?** |
| 1 (Not very confident) – 5 (Very confident) |
| **To what extent are you worried that an ONLINE format of teaching ISCE may affect your overall performance in a real ISCE examination.** |
| 1 (Not worried at all) – 5 (Extremely worried) |
| **What factors are you worried about being taught ISCE online (lecture and tutorial)?** |
| Asking questions |
| Listening clearly |
| Speaking in parallel |
| Surrounding factors (noise, other household members) |
| Quality of Internet |
| Unreliability of my device |
| Difficulty being rapport with tutor |
| (Textbox for others) |
| **If you DO NOT do well in an ISCE, to what extent do you think ONLINE teaching of ISCE could be a contributor?** |
| 1 (Irrelevant factor) – 5 (Very strong factor) |
| **If you DO well in the real ISCE, to what extent do you think ONLINE teaching of ISCE could be a contributor?** |
| 1 (Irrelevant factor) – 5 (Very strong factor) |
| **To what extent would an ONLINE format of ISCE teaching affect your ANXIETY in excelling in real ISCEs** |
| 1 (Much less anxious) – 5 (Much more anxious) |
| **To what extent will an ONLINE format of ISCE teaching affect my OVERALL PERFORMANCE in a real ISCE?** |
| 1 (Much more negatively) – 5 (Much more positively) |
| **A 64-year-old man presents to the emergency department with dyspnoea. He has a past medical history of COPD. On admission, a chest x-ray is taken. What is the most likely cause of his dyspnoea according to the Chest X-ray below?**  **Chest X-Ray of a patient with a COPD exacerbation*** |
| COPD exacerbation |
| Pneumonia |
| Pneumothorax |
| Cor pulmonale |
| Pulmonary Embolism |
| **An 80-year-old woman presents with shortness of breath. An ABG is performed and the results are shown. What does the ABG show?**  **PaO_2_: 11 kPa (11-13 kPa)**  **pH: 7.50 (7.35 – 7.45)**  **PaCO_2_: 2.6 kPa (4.7 – 6.0 kPa)**  **HCO_3_^-^: 25 (22 – 26 mEq/L)**  **BE: 1 (-2 to +2)** |
| Metabolic alkalosis with partial respiratory compensation |
| Type 2 Respiratory Failure |
| Respiratory alkalosis with partial metabolic compensation |
| Respiratory alkalosis with no metabolic compensation |
| Respiratory acidosis with partial metabolic compensation |
| **An 88-year-old man presents to the emergency department with features highly suggestive of a COPD exacerbation. Which of the following is NOT a typical feature of COPD?** |
| Chest pain |
| Long history of smoking |
| Productive Cough |
| Shortness of breath |
| Wheeze |
| **A 38-year-old woman develops pleuritic chest pain. She has been in the recovery ward for the past two days after having a total hip replacement. The surgical registrar suspects the patient has a pulmonary embolism. Which of the following is most likely to be associated with pulmonary embolism?** |
| Chest pain worse lying flat |
| Productive Cough |
| Dry Cough |
| Haemoptysis |
| High-grade fever |
| **A 76-year-old patient presents with shortness of breath. She has a history of rheumatoid arthritis. On spirometry, she has a significantly reduced forced vital capacity (FVC) compared to her predicted value. Which of the following is the likely diagnosis?** |
| Idiopathic Pulmonary Fibrosis |
| Pleural Effusion |
| COPD |
| Asthma |
| Malnutrition |
| **You are conducting a respiratory examination on a patient with confirmed pneumonia in his left lung. Which of the following findings would be expected on examination?** |
| Increased breath sounds |
| Hyper-resonance to percussion |
| Course Crackles |
| Tracheal Deviation |
| Decreased vocal resonance |
| **Which of the following is NOT a cause of digital clubbing?** |
| Bronchiectasis |
| Lung Cancer |
| Chronic Lung Abscess |
| COPD |
| Empyema |
| **Which of the following interventions will improve the prognosis of a patient with COPD the most?** |
| Long term oxygen therapy |
| Initiating SABA/SAMA therapy |
| Initiating LABA/LAMA therapy |
| Smoking Cessation |
| Nebulised salbutamol |
| **When explaining inhaler technique, how long should you tell the patient to hold their breath for after pressing the canister on the inhaler?** |
| 5 seconds |
| 10 seconds |
| 15 seconds |
| 30 seconds |
| 60 seconds |
| **You measure a patient's peak flow three times. Her readings were 500,560 and 530 litres per minute. What is her final peak flow reading?** |
| 500 litres per minute |
| 530 litres per minute |
| 550 litres per minute |
| 560 litres per minute |
| 1590 litres per minute |

*Pre-Gastroenterology Quiz*

Each quiz is started by common questions followed by case-based SBAs.

| **A 68 year old patient has recently been diagnosed with chronic mesenteric ischaemia. Which of the following is likely to be in line with the history he would have described at presentation.** |
| --- |
| Severe abdominal pain 4-5 hours after eating |
| Umbilical pain which then radiates down to the right iliac fossa |
| Severe pain which increases in increases in intensity then decreases- 'comes in waves |
| Severe abdominal pain, upon eating, which goes down a few hours after the meal |
| Severe pain, radiating to the back, associated with vomiting |

| **A 25 year old patient presents with severe diarrhoea and lower abdominal pain. Which of the following, if mentioned in the history points towards a diagnosis of Ulcerative colitis?** |
| --- |
| Blood around the pan, after defecation |
| Bloody diarrhoea |
| Severe abdominal pain, localised to the left lower quadrant |
| Severe abdominal pain, which is worse in the middle of the patients menstrual cycle |
| Severe pain on defecation. |
| **When taking blood cultures which of the following is an essential step?** |
| Wipe the area of the vein in a 'Hashtag' pattern |
| Take the blood sample from an artery |
| Fill the Aerobic bottle first then the anaerobic bottle |
| Fill the blood into syringe and then decant it into the bottles |
| Do not use a tourniquet for blood cultures |
| **A 76 year old female patient presents with severe pain, which she describes is worse in the left lower side of her abdomen. She has a temperature of 38.5 degrees celcius. She has a past medical history of hypertension, diabetes and constipation for which she has had to come to hospital to take an enema on two previous occasions. Based on the above what is the most likely diagnosis?** |
| Appendicitis |
| Diverticulitis |
| Mesenteric Infarction |
| Ovarian Torsion |
| Polycystic Kidney disease |
| **State the explanation behind these results.**  **Picture of hepatitis B antigens *** |
| Acute Hep B infection |
| Chronic Hep B infection |
| Cleared Hep B infection |
| Previously vaccinated against Hep B infection |
| Acute Hep C infection |
| **You are asked to administer a subcutaneous injection of adalimumab for a patient suffering from Crohn's disease. What colour needle is most suitable for this?** |
| Green |
| Blue |
| Black |
| Orange |
| Purple |
| **A 44 year old lady presents with severe pain in the right upper quadrant. She has a temperature of 38.3 degrees celcius and a BMI of 31. Which of the following signs is most likely to be seen in this patient.** |
| Murphy's sign |
| Cullen's sign |
| Double duct sign |
| Grey-Turner's sign |
| Rosving's sign |
| **A patient with severe right iliac fossa pain, which started as peri-umbilical before radiates down to the right iliac fossa. She is diagnosed with acute appendicitis. Which of the following signs is most likely to be found on examination of this patient.** |
| Coffee bean sign |
| Cullen's sign |
| Double duct sign |
| Grey-Turner's sign |
| Rosving's sign |
| **What condition does this abdominal x-ray most likely suggest?**  **Image of abdominal x-ray*** |
| Ischemic colitis |
| Small bowel perforation |
| Large bowel perforation |
| Sigmoid volvulus |
| Toxic megacolon |
| **Bob is a 35-year-old gentlemen who came into the clinic with fatigue, arthritis and complains of erectile dysfunction. On observation, you have noticed that his skin has a bronze colour to it. Blood test results came back with increased transferrin, ferritin and Fe and decreased TIBC. State the first line management for Bob.** |
| Desferrioxamine |
| Iron Supplements |
| Penicillamine |
| Venesection |
| Viagra |

*Post-gastroenterology quiz*

Each quiz is started by common questions followed by case-based SBAs.

| **Gender?** |
| --- |
| Male |
| Female |
| Other |
| Prefer not to Say |
| **Age?** |
| (Text box) |
| **How confident do you feel being taught ISCE (quality of teaching) using an ONLINE format?** |
| 1 (Not very confident) – 5 (Very confident) |
| **To what extent are you worried that an ONLINE format of teaching ISCE may affect your overall performance in a real ISCE examination.** |
| 1 (Not worried at all) – 5 (Extremely worried) |
| **What factors are you worried about being taught ISCE online (lecture and tutorial)?** |
| Asking questions |
| Listening clearly |
| Speaking in parallel |
| Surrounding factors (noise, other household members) |
| Quality of Internet |
| Unreliability of my device |
| Difficulty being rapport with tutor |
| (Textbox for others) |
| **If you DO NOT do well in an ISCE, to what extent do you think ONLINE teaching of ISCE could be a contributor?** |
| 1 (Irrelevant factor) – 5 (Very strong factor) |
| **If you DO well in the real ISCE, to what extent do you think ONLINE teaching of ISCE could be a contributor?** |
| 1 (Irrelevant factor) – 5 (Very strong factor) |
| **To what extent would an ONLINE format of ISCE teaching affect your ANXIETY in excelling in real ISCEs** |
| 1 (Much less anxious) – 5 (Much more anxious) |
| **To what extent will an ONLINE format of ISCE teaching affect my OVERALL PERFORMANCE in a real ISCE?** |
| 1 (Much more negatively) – 5 (Much more positively) |
| **A 60 year old female patient presents to A&E with acute abdominal pain that came on a few hours ago and vomiting. On examination, the abdomen is distended and there is guarding. Patient has history of IBD. Patient appears very unwell. An AXR is done and is shown below. What does it show?**  **Abdominal X-Ray of a patient*** |
| Rigler sign |
| Double duct sign |
| Pneumoperitoneum |
| Falciform sign |
| Football Sign |
| **You are a junior doctor on ward where you are told to examine a 23 year-old patient. You note that the patient seems quite confused and talks about imaginary things that are not there. The patient has deranged LFTs. You suspect a diagnosis of Wilson's disease, which of the following would you most likely see on examination of this patient?** |
| Corneal arcus |
| Finger clubbing |
| Apthous ulceration |
| Kayser-Fleischer rings |
| Caput medusae |
| **A consultant asks a junior doctor to do a digital rectal examination on a 48 year old male patient. Before inserting your finger, while examining the perianal region, you ask the patient to cough to confirm a certain pathology. What pathology will be elicited?** |
| Internal haemorrhoids |
| External haemorrhoids |
| Anal fistula |
| Skin tags |
| Anal fissure |
| **You are a F1 and you have been asked to perform a full abdominal examination on Bob, a 55 year-old gentle man with long history of smoking .He has a palpable mass in the epigastric region. He also reports change in bowel habits and appetite. You suspect gastric cancer which lymph node is most likely to be enlarged?** |
| Virchow, left supraventricular |
| Virchow, right supraventricular |
| Virchow, periumbilical |
| Sister Mary Joseph, left supraventricular |
| Sister Mary Joseph, right supraventricular |
| **Please interpret the following results.**  **Image showing hepatitis B antigen*** |
| Previous Hep B vaccination |
| Acute Hep B infection |
| Previous Hep B infection, now immune |
| Chronic Hep B infection |
| Chronic Hep D co-infection |
| **A patient complains of PR bleeding. Which of the following if mentioned in the history will point towards a diagnosis of haemorrhoids.** |
| Black, smelly tarry stools |
| Blood around the toilet pan |
| Extreme pain on defecation |
| Mucus mixed in stool |
| Pale stool, which is difficult to flush |
| **Which of the following if mentioned in the patient's history will most points towards a diagnosis of IBS.** |
| Abdominal pain, worse in the left iliac fossa |
| Abdominal pain worse on defecation |
| Abdominal pain better on defecation |
| Abdominal distention with fluid thrills |
| Abdominal pain, hepatomegaly and ascites |
| **You are asked to place a cannula in a patient who will require a blood transfusion, post a small bowel resection. What colour cannula is best suited for this purpose?** |
| Blue |
| Green |
| Pink |
| Purple |
| Orange |
| **A 85 year old patient presents with sudden on set severe abdominal pain. An ECG shows the patient is in AF what is the most likely diagnosis?** |
| Ruptured AAA |
| Bowel perforation |
| Biliary sepsis |
| Ischaemic colitis |
| Mesenteric Ischaemia |
| **A 31 year old male presents to the GP with excessive diarrhoea, loss of weight, changes in appetite. He reports having to go 7-8 times a day. He describes that the diarrhoea contains mucus with blood being present. Given the most likely diagnosis, what is the most appropriate drug to maintain remission?** |
| Methotrexate |
| Azathioprine |
| Mesalazine |
| Aspirin |
| Infliximab |

*Pre-Neurology Quiz*

Each quiz is started by common questions followed by case-based SBAs.

| **A 26-year-old woman presents to the emergency department with poor vision in the left eye. She also has painful eye movements. 6 months ago, she suffered from urinary incontinence which eventually resolved. An MRI Head is performed. What is the likely diagnosis?** |
| --- |
| Diabetic neuropathy |
| Ischaemic Stroke |
| Multiple Sclerosis |
| Cerebellar syndrome |
| B12 deficiency |

| **A 57 year old man presents to the emergency department after recent trauma. A CT Head is performed. What does the CT head show?**  **CT head image shown*** |
| --- |
| No abnormalities |
| Acute subdural haematoma |
| Ischaemic Stroke |
| Subarachnoid haemorrhage |
| Extradural haemorrhage |
| **A 65-year-old woman is brought in by ambulance with weakness. She has reduced power in her arms compared to her legs. The junior doctor suspect the patient has an occlusion of her left middle cerebral artery. What other symptoms might be expected in this patient?** |
| Aphasia |
| Right homonymous hemianopia with macular sparing |
| Right sided loss of pain and temperature |
| Left homonymous hemianopia without macular sparing |
| Nystagmus |
| **A 64-year-old is brought in by ambulance after he noticed left sided weakness which started 3 hours ago. He has a past medical history of atrial fibrillation. What is the first line investigation?** |
| MRI Head |
| Nerve Conduction Studies |
| CT Head |
| Echocardiogram |
| CT Angiogram |
| **Which structure is labelled with the yellow cross on this MRI Head?**  **MRI head image shown*** |
| Pituitary gland |
| Corpus Callosum |
| Third Ventricle |
| Thalamus |
| Sphenoid sinus |
| **On examination, a patient has increased tone in her legs with spasticity and absent ankle jerks. She has reduced pin prick sensation in the feet and lower legs, and Romberg's sign is positive. Which of the following is the likely diagnosis?** |
| Subacute combined degeneration of the spinal cord |
| Syringomyelia |
| Anterior spinal cord syndrome |
| Multiple Sclerosis |
| Stroke |
| **Which of the following dermatomes corresponds to the big toe?** |
| L3 |
| L4 |
| L5 |
| S1 |
| S2 |
| **A 58 year old man with a history of alcohol excess presents with confusion and an unstable gait. On examination, he has bilateral nystagmus. What is the most appropriate initial treatment for this patient?** |
| Intravenous glucose |
| Intravenous thiamine |
| Intravenous B12 |
| Intravenous folate |
| Intravenous terlipressin |
| **Which of the following is essential to mention when you talk to a patient before performing a capillary glucose measurement?** |
| Ask if the patient has a history of bleeding |
| Ask if the patient has a history of diabetes |
| Ask the patient to wash hands |
| Ask if the patient is on anticoagulants |
| Ask the patient to squeeze their finger to encourage blood flow |
| **When performing a capillary glucose measurement, which of the following must you NOT do?** |
| Apply the test strip on the first drop of drop |
| Place gauze over the puncture site after taking the measurement |
| Dispose the lancet into a sharps bin |
| Choose the medial/lateral border of the finger pulp |
| Massage the finger from proximal to distal to encourage blood flow |

*Post-neurology quiz*

Each quiz is started by common questions followed by case-based SBAs.

| **Gender?** |
| --- |
| Male |
| Female |
| Other |
| Prefer not to Say |
| **Age?** |
| (Text box) |
| **How confident do you feel being taught ISCE (quality of teaching) using an ONLINE format?** |
| 1 (Not very confident) – 5 (Very confident) |
| **To what extent are you worried that an ONLINE format of teaching ISCE may affect your overall performance in a real ISCE examination.** |
| 1 (Not worried at all) – 5 (Extremely worried) |
| **What factors are you worried about being taught ISCE online (lecture and tutorial)?** |
| Asking questions |
| Listening clearly |
| Speaking in parallel |
| Surrounding factors (noise, other household members) |
| Quality of Internet |
| Unreliability of my device |
| Difficulty being rapport with tutor |
| (Textbox for others) |
| **If you DO NOT do well in an ISCE, to what extent do you think ONLINE teaching of ISCE could be a contributor?** |
| 1 (Irrelevant factor) – 5 (Very strong factor) |
| **If you DO well in the real ISCE, to what extent do you think ONLINE teaching of ISCE could be a contributor?** |
| 1 (Irrelevant factor) – 5 (Very strong factor) |
| **To what extent would an ONLINE format of ISCE teaching affect your ANXIETY in excelling in real ISCEs** |
| 1 (Much less anxious) – 5 (Much more anxious) |
| **To what extent will an ONLINE format of ISCE teaching affect my OVERALL PERFORMANCE in a real ISCE?** |
| 1 (Much more negatively) – 5 (Much more positively) |
| **Which of the following nerve roots represent the supply to the anterior arm and radial side of hand (thumb and index finger)?** |
| C4 |
| C5 |
| C6 |
| C7 |
| C8 |
| **A 25 year-old male was assaulted on his way home after a night out in Juice. On examination, you observed the following sign. What is the name of this sign?**  **Image of battle sign*** |
| Battle's sign |
| Ramsey's sign |
| Crescent's sign |
| Hunter's sign |
| Turner's sign |
| **The following MRI image shows a sagittal section of the brain. What does the letter D correspond to?** **The following MRI image shows a sagittal section of the brain. What does the letter D correspond to?**  **Image of MRI brain with labels*** |
| Corpus callosum |
| Thalamus |
| Pituitary gland |
| Medulla |
| Pons |
| **A 5 year-old boy fell off a ladder while playing with his siblings. On examination, you saw the following observation. State the most likely reason behind this observation.**  **Image of racoon eyes*** |
| Basal skull fracture |
| Dislodged eye socket |
| Cellulitis of the eye lids |
| Subconjunctival bleeding |
| Frontal skull fracture |
| **A 23 year old female comes in complaining of severe left sided headache which has resulted in her taking several days off work. She complains of a throbbing sensation during these episodes. The doctor suspects a migraine. Given the diagnosis, what is another feature you would expect?** |
| Unilateral Lacrimation |
| Unilateral deafness |
| Restlessness |
| Electric shock like pain around eye |
| Going into a dark room to sleep |
| **An 80 year old women presents to A&E with a GCS of 13/15, chronic headache, weakness in the left arm and speech difficulties. A CT scan is done which shows a hypodense region with a concave shape and is not restricted to suture lines. What is the most likely diagnosis?** |
| Extradural Haemorrhage |
| Subdural Haemorrhage |
| Subarachnoid Haemorrhage |
| Ischaemic stroke |
| Haemorrhagic stroke |
| **A 58 year old male patient comes into A&E with confusion, fever, sudden worsening of his chronic headache and episodes of vomiting. A CT head is done to investigate. What does the CT scan show?**  **Image of CT head with ring-enhanced lesion*** |
| Space occupying lesion |
| Intracranial haemorrhage |
| Cerebral abcess |
| Cerebral infarction |
| Radiation necrosis |
| **A rugby player presents with a sudden thunderclap headache after a horrendous high tackle by an opposite player. He also notices neck stiffness. When taking a history from this patient, which of the following is a risk factor for this condition?** |
| Hypertrophic obstructive cardiomyopathy |
| Turner syndrome |
| Down syndrome |
| Polycystic Kidney Disease |
| Coarctation of aorta |
| **A male patient presented with episodes of headaches. He is a heavy smoker and drinks 30 pints a week. His headaches last around 30 minutes twice a day and had been going on for the last 10 weeks. He had a feeling of intense sharp pain around one eye during his episodes of headaches. He is feeling fine now. Given the most likely diagnosis, State the most appropriate prophylaxis to manage his condition.** |
| Paracetamol |
| Triptan |
| Oxygen |
| Propranolol |
| Verapamil |
| **A year 2 medical student is asked to explain how to carryout venepuncture on a simulated patient. Which of following statements in her explanation is incorrect?** |
| Palpate the vein before wiping with alcohol swab |
| Remove the tourniquet after removing the needle |
| Areas of bruised skin must be avoided |
| Anchor the vein below with the non-dominant hand before insertion of needle |
| Insert the needle at a 30 degree angle to the skin surface |

Feedback forms

Cardio

| **Email** |
| --- |
| (Text box) |
| **What university do you attend?** |
| (Text box) |
| **What is your current stage of training?** |
| Year 1 |
| Year 2 |
| Year 3 |
| Year 4 |
| Year 5 |
| Intercalating |
| Other: (Text box) |
| **How would you rate the organisation of the event?** |
| 1-10 |
| **How would you rate the publicity of the event?** |
| 1-10 |
| **How would you rate the usefulness of the event?** |
| 1-10 |
| **Presenter’s (Nish) knowledge of subject matter** |
| 1-10 |
| **Presenter’s (Nish) delivery of subject matter** |
| 1-10 |
| **Presenter’s (Movin) knowledge of subject matter** |
| 1-10 |
| **Presenter’s (Movin) delivery of subject matter** |
| 1-10 |
| **Additional comments** |
| (Text box) |

Respiratory

| **Email** |
| --- |
| (Text box) |
| **What university do you attend?** |
| University of Aberdeen School of Medicine and Dentistry |
| Anglia Ruskin University School of Medicine |
| Aston University Medical School |
| Barts and The London School of Medicine and Dentistry |
| University of Birmingham College of Medical and Dental Sciences |
| Brighton and Sussex Medical School |
| University of Bristol Medical School |
| University of Buckingham Medical School |
| University of Cambridge School of Clinical Medicine |
| Cardiff University School of Medicine |
| University of Dundee School of Medicine |
| Edge Hill University Medical School |
| The University of Edinburgh Medical School |
| University of Exeter Medical School |
| University of Glasgow School of Medicine |
| Hull York Medical School |
| Imperial College London Faculty of Medicine |
| Keele University School of Medicine |
| Kent and Medway Medical School |
| King's College London GKT School of Medical Education |
| Lancaster University Medical School |
| University of Leeds School of Medicine |
| University of Liverpool School of Medicine |
| London School of Hygiene & Tropical Medicine |
| University of Manchester Medical School |
| Newcastle University School of Medical Education |
| Norwich Medical School |
| University of Nottingham School of Medicine |
| University of Nottingham - Lincoln Medical School |
| University of Oxford Medical Sciences Division |
| Plymouth University Peninsula Schools of Medicine and Dentistry |
| Queen's University Belfast School of Medicine |
| University of Sheffield Medical School |
| University of Southampton School of Medicine |
| University of St Andrews School of Medicine |
| St George's, University of London |
| University of Sunderland School of Medicine |
| Swansea University Medical School |
| University of Central Lancashire School of Medicine |
| University College London Medical School |
| University of Warwick Medical School |
| Other: (Text box) |
| **What is your current stage of training?** |
| Year 1 |
| Year 2 |
| Year 3 |
| Year 4 |
| Year 5 |
| Intercalating |
| Other: (Text box) |
| **How would you rate the organisation of the event?** |
| 1-10 |
| **How would you rate the publicity of the event?** |
| 1-10 |
| **How would you rate the usefulness of the event?** |
| 1-10 |
| **Presenter’s (Nish) knowledge of subject matter** |
| 1-10 |
| **Presenter’s (Nish) delivery of subject matter** |
| 1-10 |
| **Presenter’s (Movin) knowledge of subject matter** |
| 1-10 |
| **Presenter’s (Movin) delivery of subject matter** |
| 1-10 |
| **Additional comments** |
| (Text box) |

Gastroenterology

| **Email** |
| --- |
| (Text box) |
| **What university do you attend?** |
| University of Aberdeen School of Medicine and Dentistry |
| Anglia Ruskin University School of Medicine |
| Aston University Medical School |
| Barts and The London School of Medicine and Dentistry |
| University of Birmingham College of Medical and Dental Sciences |
| Brighton and Sussex Medical School |
| University of Bristol Medical School |
| University of Buckingham Medical School |
| University of Cambridge School of Clinical Medicine |
| Cardiff University School of Medicine |
| University of Dundee School of Medicine |
| Edge Hill University Medical School |
| The University of Edinburgh Medical School |
| University of Exeter Medical School |
| University of Glasgow School of Medicine |
| Hull York Medical School |
| Imperial College London Faculty of Medicine |
| Keele University School of Medicine |
| Kent and Medway Medical School |
| King's College London GKT School of Medical Education |
| Lancaster University Medical School |
| University of Leeds School of Medicine |
| University of Liverpool School of Medicine |
| London School of Hygiene & Tropical Medicine |
| University of Manchester Medical School |
| Newcastle University School of Medical Education |
| Norwich Medical School |
| University of Nottingham School of Medicine |
| University of Nottingham - Lincoln Medical School |
| University of Oxford Medical Sciences Division |
| Plymouth University Peninsula Schools of Medicine and Dentistry |
| Queen's University Belfast School of Medicine |
| University of Sheffield Medical School |
| University of Southampton School of Medicine |
| University of St Andrews School of Medicine |
| St George's, University of London |
| University of Sunderland School of Medicine |
| Swansea University Medical School |
| University of Central Lancashire School of Medicine |
| University College London Medical School |
| University of Warwick Medical School |
| Other: (Text box) |
| **What is your current stage of training?** |
| Year 1 |
| Year 2 |
| Year 3 |
| Year 4 |
| Year 5 |
| Intercalating |
| Other |
| **How would you rate the organisation of the event?** |
| 1-10 |
| **How would you rate the publicity of the event?** |
| 1-10 |
| **How would you rate the usefulness of the event?** |
| 1-10 |
| **Presenter’s (Nish) knowledge of subject matter** |
| 1-10 |
| **Presenter’s (Nish) delivery of subject matter** |
| 1-10 |
| **Presenter’s (Srinjay) knowledge of subject matter** |
| 1-10 |
| **Presenter’s (Srinjay) delivery of subject matter** |
| 1-10 |
| **Additional comments** |
| (Text box) |

Neurology

| **Email** |
| --- |
| (Text box) |
| **What university do you attend?** |
| University of Aberdeen School of Medicine and Dentistry |
| Anglia Ruskin University School of Medicine |
| Aston University Medical School |
| Barts and The London School of Medicine and Dentistry |
| University of Birmingham College of Medical and Dental Sciences |
| Brighton and Sussex Medical School |
| University of Bristol Medical School |
| University of Buckingham Medical School |
| University of Cambridge School of Clinical Medicine |
| Cardiff University School of Medicine |
| University of Dundee School of Medicine |
| Edge Hill University Medical School |
| The University of Edinburgh Medical School |
| University of Exeter Medical School |
| University of Glasgow School of Medicine |
| Hull York Medical School |
| Imperial College London Faculty of Medicine |
| Keele University School of Medicine |
| Kent and Medway Medical School |
| King's College London GKT School of Medical Education |
| Lancaster University Medical School |
| University of Leeds School of Medicine |
| University of Liverpool School of Medicine |
| London School of Hygiene & Tropical Medicine |
| University of Manchester Medical School |
| Newcastle University School of Medical Education |
| Norwich Medical School |
| University of Nottingham School of Medicine |
| University of Nottingham - Lincoln Medical School |
| University of Oxford Medical Sciences Division |
| Plymouth University Peninsula Schools of Medicine and Dentistry |
| Queen's University Belfast School of Medicine |
| University of Sheffield Medical School |
| University of Southampton School of Medicine |
| University of St Andrews School of Medicine |
| St George's, University of London |
| University of Sunderland School of Medicine |
| Swansea University Medical School |
| University of Central Lancashire School of Medicine |
| University College London Medical School |
| University of Warwick Medical School |
| Other: (Text box) |
| **What is your current stage of training?** |
| Year 1 |
| Year 2 |
| Year 3 |
| Year 4 |
| Year 5 |
| Intercalating |
| Other: (Text box) |
| **How would you rate the organisation of the event?** |
| 1-10 |
| **What type of teaching would you like us to focus on in May? (Tick all that apply)** |
| OSCEs |
| Finals |
| Other: (Text box) |
| **How would you rate the publicity of the event?** |
| 1-10 |
| **How would you rate the usefulness of the event?** |
| 1-10 |
| **Presenter’s (Hallam) knowledge of subject matter** |
| 1-10 |
| **Presenter’s (Hallam) delivery of subject matter** |
| 1-10 |
| **Presenter’s (Ravanth) knowledge of subject matter** |
| 1-10 |
| **Presenter’s (Ravanth) delivery of subject matter** |
| 1-10 |
| **Additional comments** |
| (Text box) |
