# Supplementary material for: Enhancing medical students` confidence and performance in integrated structured clinical examinations (ISCE) through a novel near-peer, mixed model approach during the COVID-19 pandemic
Source: BMC Med Educ. 2023 Feb 23;23:128. doi: 10.1186/s12909-022-03970-y (PMC9947444; doi:10.1186/s12909-022-03970-y)
Supplement: Supplementary file 2 — Additional file 2. [file 12909_2022_3970_MOESM2_ESM.docx]

**Analysed Raw Data**

Precardio 44.44% (29.75%)+ 74.83% (16.52%)

Prerespiratory 54.38% (30%)+ 71.43% (12.78%)

Pregastro 57.85% (24.74%) + 52.79% (26.6%)

Preneuro 75.69% (18.27%)+ 72.14 (25.76%)

Proportions Correct

X-squared = 107.93, df = 1, p-value < 2.2e-16

alternative hypothesis: two.sided

95 percent confidence interval:

-0.2019944 -0.1386820

sample estimates:

prop 1(Before) prop 2 (After)

0.5372642 0.7076023

summary(X$Age)

Min. 1st Qu. Median Mean 3rd Qu. Max. NA's

1.00 21.00 23.00 24.38 25.00 60.00 4

> X %>%

+ count(X$Gender, sort = TRUE)

X$Gender n

1 Female 56

2 Male 16

> X %>%

+ count(X$Have.you.attended.any.previous.ONLINE.ISCE.OSCE.teaching., sort = TRUE)

X$Have.you.attended.any.previous.ONLINE.ISCE.OSCE.teaching. n

1 No 64

2 Yes 8

summary(X$How.confident.do.you.feel.being.taught.ISCE..quality.of.teaching..using.an.ONLINE.format.)

Min. 1st Qu. Median Mean 3rd Qu. Max.

1.000 3.000 3.000 3.444 4.000 5.000

> summary(X$How.confident.do.you.feel.being.taught.ISCE..quality.of.teaching..IN.PERSON.)

Min. 1st Qu. Median Mean 3rd Qu. Max.

3.000 4.000 4.500 4.486 5.000 5.000

> wilcox.test(X$How.confident.do.you.feel.being.taught.ISCE..quality.of.teaching..using.an.ONLINE.format.,X$How.confident.do.you.feel.being.taught.ISCE..quality.of.teaching..IN.PERSON.)

Wilcoxon rank sum test with continuity correction

data: X$How.confident.do.you.feel.being.taught.ISCE..quality.of.teaching..using.an.ONLINE.format. and X$How.confident.do.you.feel.being.taught.ISCE..quality.of.teaching..IN.PERSON.

W = 840, p-value = 1.104e-13

alternative hypothesis: true location shift is not equal to 0

**TEACHERS Perception**

summary(X$How.confident.did.you.feel.teaching.ISCE.content.using.an.online.format.BEFORE.our.OSCEazy.program.)

Min. 1st Qu. Median Mean 3rd Qu. Max. NA's

1.000 2.000 3.000 2.893 3.250 5.000 44

> summary(X$How.confident.do.you.feel.teaching.ISCE.content.using.an.online.format.now.AFTER.our.OSCEazy.program.)

Min. 1st Qu. Median Mean 3rd Qu. Max. NA's

3.000 4.000 4.000 4.286 5.000 5.000 44

> wilcox.test(X$How.confident.did.you.feel.teaching.ISCE.content.using.an.online.format.BEFORE.our.OSCEazy.program.,X$How.confident.do.you.feel.teaching.ISCE.content.using.an.online.format.now.AFTER.our.OSCEazy.program.)

Wilcoxon rank sum test with continuity correction

data: X$How.confident.did.you.feel.teaching.ISCE.content.using.an.online.format.BEFORE.our.OSCEazy.program. and X$How.confident.do.you.feel.teaching.ISCE.content.using.an.online.format.now.AFTER.our.OSCEazy.program.

W = 102, p-value = 6.605e-07

alternative hypothesis: true location shift is not equal to 0

> summary(X$How.confident.did.you.feel.teaching.ISCE.content.in.person.BEFORE.our.OSCEazy.program.)

Min. 1st Qu. Median Mean 3rd Qu. Max. NA's

2.000 2.000 3.000 3.036 4.000 5.000 44

> summary(X$How.confident.do.you.feel.teaching.ISCE.content.in.person.AFTER.our.OSCEazy.program.)

Min. 1st Qu. Median Mean 3rd Qu. Max. NA's

3.000 3.000 4.000 3.929 4.000 5.000 44

> wilcox.test(X$How.confident.did.you.feel.teaching.ISCE.content.in.person.BEFORE.our.OSCEazy.program.,X$How.confident.do.you.feel.teaching.ISCE.content.in.person.AFTER.our.OSCEazy.program.)

Wilcoxon rank sum test with continuity correction

data: X$How.confident.did.you.feel.teaching.ISCE.content.in.person.BEFORE.our.OSCEazy.program. and X$How.confident.do.you.feel.teaching.ISCE.content.in.person.AFTER.our.OSCEazy.program.

W = 196, p-value = 0.0008701

alternative hypothesis: true location shift is not equal to 0

> summary(X$precardio_confidence_online.)

Min. 1st Qu. Median Mean 3rd Qu. Max. NA's

1.000 3.000 3.000 3.444 4.000 5.000 2048

> summary(X$postcardio_confidence_online.)

Min. 1st Qu. Median Mean 3rd Qu. Max. NA's

2.000 3.000 4.000 3.727 4.000 5.000 2054

> wilcox.test(X$precardio_confidence_online.,X$postcardio_confidence_online.)

Wilcoxon rank sum test with continuity correction

data: X$precardio_confidence_online. and X$postcardio_confidence_online.

W = 1856.5, p-value = 0.01541

alternative hypothesis: true location shift is not equal to 0

>

> summary(X$precardio_anxiety_online.)

Min. 1st Qu. Median Mean 3rd Qu. Max. NA's

1.000 2.000 3.000 3.014 4.000 5.000 2048

> summary(X$postcardio_anxiety_online.)

Min. 1st Qu. Median Mean 3rd Qu. Max. NA's

1.000 3.000 3.000 3.151 4.000 5.000 2054

> wilcox.test(X$precardio_anxiety_online.,X$postcardio_anxiety_online.)

Wilcoxon rank sum test with continuity correction

data: X$precardio_anxiety_online. and X$postcardio_anxiety_online.

W = 2173.5, p-value = 0.3672

alternative hypothesis: true location shift is not equal to 0

>

> summary(X$precardio_overall.performnace_online.)

Min. 1st Qu. Median Mean 3rd Qu. Max. NA's

1.000 3.000 3.000 3.444 4.000 5.000 2048

> summary(X$postcardio_overall.performnace_online.)

Min. 1st Qu. Median Mean 3rd Qu. Max. NA's

2.000 3.000 3.000 3.242 4.000 5.000 2054

> wilcox.test(X$precardio_overall.performnace_online.,X$postcardio_overall.performnace_online.)

Wilcoxon rank-sum test with continuity correction

data: X$precardio_overall.performnace_online. and X$postcardio_overall.performnace_online.

W = 2734, p-value = 0.09736

alternative hypothesis: true location shift is not equal to 0
