# Supplementary material for: Enhancing medical students` confidence and performance in integrated structured clinical examinations (ISCE) through a novel near-peer, mixed model approach during the COVID-19 pandemic
Source: BMC Med Educ. 2023 Feb 23;23:128. doi: 10.1186/s12909-022-03970-y (PMC9947444; doi:10.1186/s12909-022-03970-y)
Supplement: Supplementary file 3 — Additional file 3. [file 12909_2022_3970_MOESM3_ESM.docx]

**Participant Information Sheet**

You are invited to take part in a research study conducted by students at Cardiff University. Before you decide, it is important for you to understand why the research is being done and what it will involve. Please take time to read the following information carefully and discuss it with others if you wish. Please do not hesitate to contact the researchers if anything is unclear or if you would like further information. Please take time to decide whether or not you wish to participate.

Thank you for taking the time to read this.

**Study title**:

**To assess the effectiveness of a virtual multimodal OSCE teaching and it’s effect on student confidence and competence.**

You are being invited to take part in a research project. Before you decide whether or not to take part it is important for you to understand why the research is being undertaken and what it will involve **and how you will be involved**. Please take time to read the following information carefully and discuss it with others, if you wish.

Thank you for reading this.

**What is the purpose of the study?**

The OSCE teaching series, OSCEazy founded by senior Cardiff University medical students aims to deliver quality virtual near peer mentoring OSCE teaching to second year Cardiff University medical students.

This longitudinal study aims to evaluate the effectiveness of a multimodal virtual OSCE revision series on improving student confidence and competence.

To help with this, we have created a secure online questionnaire on google forms, under the account of [isce1to2@gmail.com](mailto:isce1to2@gmail.com), which can only be accessed by the main authors of the study.

The research will take place over a 3-month period from 16^th^ April 2021 to 13^th^ July 2021.

**What is involved in participating in the research/study?**

Taking part in this study involves completing a secured online questionnaire on google forms.

**Why have I been invited to take part?**

You have signed up for a multimodal virtual OSCE revision series, and by evaluating your perceptions and performance, the efficacy of this method of teaching can be determined.

**Do I have to take part?**

## Taking part is entirely voluntary and is up to you to decide whether or not to take part.

By submitting this form, you are providing us consent to use the data you have given us in this study.

**Will I be paid for taking part?**

No.

You should understand that any data you give will be as a gift and will not benefit you financially in the future should this research project lead to the development of a new method.

**What about confidentiality?**

There is no identifiable data being collected in the questionnaire. All information gathered from the questionnaire will be stored securely. No person will be identifiable in any report or publication of the study results.

**What will happen to my Personal Data?**

There is no identifiable data being collected in the questionnaire. Information will be held in confidence in accordance with General Data Protection Regulation (GDPR) principles in the UK. (according to Article 6(1)(a) of the GDPR).

Data on the google form will be held for a period of 1 year for the purposes of comparing trends and analytics according to the Cardiff University guidance. Access to the data is only granted to the main authors. The data will be used for academic publications and will be stored in a password-protected account.

**What are the benefits and risks in participating in this research study?**

The study will help evaluate the efficacy of virtual OSCE teaching as well as near peer mentoring in improving OSCE confidence and competence which will be valuable in designing a future teaching series.

This is a voluntary questionnaire. There are no particular risks to health. However, any person whose illness could worsen by filling in the questionnaire is advised not to volunteer. If you experience any change in your physical or emotional health, we advise you contact your medical practitioner as you would normally.

**What will happen to the results of the research?**

Data will be held for a period of 1 year for the purposes of comparing trends and analytics. Access to the data is only granted to the main authors. The data will be used for academic publications and will be stored in a password-protected account.

Any questions, concerns or requests regarding the use of data can be directed to: isceazy1to2@gmail.com

**What if there is a problem?**

If you wish to complain, or have grounds for concerns about any aspect of the manner in which you have been approached or treated during the course of this research, please contact the steering committee via email ([isceazy1to2@gmail.com](mailto:healthcareinternationalsociety@cardiff.ac.uk)) at any time should they wish to raise a complaint. If you feel that your complaint has not been handled to your satisfaction, you may contact someone independent from the research team such as this project’s main supervisor: Dr. Paul Brennan (brennan.p@cardiff.ac.uk)

**Who is organising and funding this research project?**

This research is organized by Cardiff University medical students: Ravanth Baskaran, Srinjay Mukhopadhyay, Movin Peramuna Gamage, Vincent Ng, Nishaanth Dalavaye, Sashiananthan Ganesananthan, Allen Mathew. Supervised by Dr. Paul Brennan.

**Who is the Data Controller and how can I contact them?**

OSCEazy group is the data controller for this project. They can be contacted at: isce1to2@gmail.com

**What are the purposes and lawful basis of processing my data?**

Under data protection law, we must specify the legal bases that we are relying on to process your personal data. In providing your personal data for this research we will process it on the basis that doing so is necessary for our public task for scientific and historical purposes in accordance with the necessary safeguards and is in the public interest. The University is a public research institution established by Royal Charter to advance knowledge and education to work teaching and research activities. Our charter can be found on the Cardiff University website [www.cardiff.ac.uk](http://www.cardiff.ac.uk)

**Who are the recipients of my personal data?**

The anonymized questionnaire results with the steering committee of OSCEazy.

**How will my personal data be stored and for what period of time?**

Your information will be stored on a password-protected account on Google forms, managed by the steering committee of OSCEazy. This anonymous information will be kept for a period of 1 year and may be published in support of the research.

**What are my rights?**

You have a number of rights under the data protection law and can find out more about these on our website. Please note that your rights to access, change or move your personal data are limited, as there will be personally identifiable information collected, due to this we cannot delete the information about you that we have already obtained.

**Who has reviewed this project?**

This research project is in the progress of being reviewed and given a favorable opinion by the School of Medicine’s Research Ethics Committee at Cardiff University. The project has also being reviewed and is supported by Dr Paul Brennan.

**Will my personal data be transferred to parties outside of the European Economic Area?**

No, your personal data will not be transferred to parties outside of the European Economic Area.

**Contact for Further Information**

If any of the information included is unclear, or you would like any additional information, please do not hesitate to contact OSCEazy via email: isceazy1to2@gmail.com

**Thank you for considering taking part in this research project. If you decide to participate, you will be given a copy of the Participant Information Sheet to keep for your records.**
