# Supplementary material for: Enhancing medical students` confidence and performance in integrated structured clinical examinations (ISCE) through a novel near-peer, mixed model approach during the COVID-19 pandemic
Source: BMC Med Educ. 2023 Feb 23;23:128. doi: 10.1186/s12909-022-03970-y (PMC9947444; doi:10.1186/s12909-022-03970-y)
Supplement: Supplementary file 4 — Additional file 4. [file 12909_2022_3970_MOESM4_ESM.pdf]

# OSCEazy

## The general format of an ISCE Station

History/ Examination

↓

Data Interpretation

↓

Clinical Skill

↓

Formative questions

They don't expect you to know that much!

A lot of it is just acting!

You MUST practice histories, examinations and clinical skills regularly!

NEVER forget to ask about allergies and what reaction they get!

Flex your knowledge!

# STRUCTURE OF A GENERAL HISTORY

INTRODUCTION → WIPE

Open questions

HISTORY OF PRESENTING COMPLAINT → SOCRATES; Systems Review

Summarise

PAST MEDICAL/ SURGICAL HISTORY → Normal Health; Risk factors

FAMILY HISTORY

DRUG HISTORY & ALLERGIES

SOCIAL HISTORY →
- **L**iving situation
- **O**ccupation
- **S**moking, alcohol & recreational drugs
- **T**ravel, hobbies, pets +/- sexual history

IDEAS, CONCERNS & EXPECTATIONS

SUMMARISE & INFORM NEXT STEPS

Empathise as appropriate

Pick up on cues

Maintain appropriate eye contact

Signpost as appropriate

# The Cardio Station

INTRODUCTION TO THE STATION

CARDIOVASCULAR HISTORY TAKING

HIGH YIELD ECGS & HOW TO PRESENT

CARDIAC BLOOD TEST & HOW TO PRESENT

CARDIOVASCULAR EXAMINATION

**ROLE:** Second Year Medical Student

**LOCATION:** Medical Admissions Unit

**SCENARIO:** You have been asked to speak to Mr/Mrs Jones who has presented with chest pain. You have 7 minutes to take a history and then you will be asked to present the history to the examiner

# INTRODUCTION

Hello, my name is .... and I am a second year medical student. Can I confirm your name and age please.

Is it okay if I take a quick history from you and then we can organise some tests to see what is causing your symptoms. Would you like some painkillers?

# Presenting complaint

## OPEN QUESTIONS

What's brought you in today?

Tell me more about the pain?

**S**ite - where is the pain?

**O**nset - Exact time? When was it worst? Do they have pain now?

**C**haracter - Crushing? Sharp? Stabbing?, Pleuritic?

**R**adiation - Jaw?, Arm?

**A**ssociated symptoms - Nausea?, Vomiting?, SOB?

**T**iming - continuous?, pattern?

**E**xcacerbating/alleviating factors - Worse on exertion?
- Better with rest?

**S**everity- Out of 10?

# SYSTEMS REVIEW

Any palpitations?
Any SOB?
Any clamminess/ nausea/vomiting?
Any syncope/ dizziness?
Any leg swelling?
Any orthopnea?
Any PND?
Any calf pain?
Any epigastric pain?

Any acid taste in mouth?
Any fever?
Any weight loss?

# SUMMARISE!!!

# Past medical/ surgical history

- Do you have any history of any medical conditions?
- Is there anything you see your GP for?
- Do you have any history of heart disease?

- Cardiovascular (Diabetes, HTN, High Cholesterol, previous MI)
- Respiratory (Immobility, long haul flight, COCP)
- Gastrointestinal (GORD)

Have you had any previous surgeries?

# Family history

- Do you have any family history of any medical conditions?

If family member died, sensitively ask at what age and how

I'm sorry to hear about that, is it okay if I can ask what medical condition was thought to have caused his/her death?

# Drug history

- Are you currently taking any medication?

- Are you taking any over the counter medication?

Key 'Chest pain' drugs

Statins

ACE-inhibitors

Beta-Blocker

Anti-coagulants

GTN spray

Calcium Channel Blockers

Antiplatelets

NSAIDs

Antibiotics

# ALLERGIES!!!

**THE MAIN WAY STUDENTS FAIL THE STATION IS NOT ASKING!!!**

Ask what happens when they take the drug

Tell the patient that you will document that allergy in his/her notes

# Social history

- I would also like to take a quick social history
- Who do you currently live? Do you have any carers?
- Do you have anyone for support?
- What type of house do you live in and do you have any adaptations to assist them?
- Are you able to do your normal daily activities independently or do they need any assistance? Any mobility aids?
- Do you regularly exercise? What type of exercise?
- Can you describe what you diet is on a normal day?

# Social history

- Do you currently smoke?
- How many packs do you smoke a day?
- When did you start smoking?

No of pack years = No of years smoked x average no packs smoked a day

- Have you considered stopping smoking? Would you like to discuss that today
- Do you drink any alcohol?
- How much alcohol would you drink in a drink? What type of alcohol?
- Do you take any recreational drugs?

# ICE

Should be DYNAMIC and should pick on verbal and non-verbal cues as appropriate.

Thank you for giving me a very comprehensive history. It would be also helpful to hear what your ideas are about what is causing your symptoms?

You must be feeling very distressed. What would you say is you biggest concern at this moment.

And so hopefully we will be help you with your symptoms today. Do you have any other expectations from us?

| Acute Coronary Syndrome | Stable Angina | Aortic Dissection |
|---|---|---|
| SEVERE CRUSHING CHEST PAIN AT REST | SIMILAR FEATURES AS ACS | SEVERE , SUDDEN ONSET TEARING CHEST PAIN |
| RADIATION TO JAW/ARM | NO PAIN AT REST | RADIATING TO THE BACK (BETWEEN SCAPULAE) |
| NAUSEA/SOB/CLAMMY/ SWEATY | SHORTER DURATION THAN ACS | SYNCOPE/ SOB |
| PAIN WORSENS BY EXERTION | GTN SPRAY IS VERY EFFECTIVE | CARDIO RISK FACTORS |
| GTN SPRAY HELPS PAIN | CARDIO RISK FACTORS | HISTORY OF MARFAN'S |
| CARDIO RISK FACTORS | | |

## Pneumothorax

PLEURITIC CHEST PAIN

SUDDEN SOB

YOUNG THIN MALE/ HISTORY OF LUNG DISEASE

## Pulmonary Embolism

PLEURITIC ‘SHARP’ CHEST PAIN

SUDDEN SOB

HAEMOPTYSIS

HISTORY OF DVT/ CALF SWELLING

HISTORY OF IMMOBILITY, ACTIVE CANCER, SURGERY, COCP OR HYPERCOAGUABLE STATE

## GORD

BURNING SENSATION BEHIND STERNUM

WORSENED BY MEALS, LYING DOWN AND STRAINING

WATER BRASH

### OTHER DIFFERENTIALS

ACUTE PERICARDITIS

PANIC ATTACK

MUSCULOSKELETAL PAIN

BOERHAAVE SYNDROME

# PRESENTING THE HISTORY

**Name, Age, Occupation & key presenting complaint:** I had the pleasure of talking to Bruce Wayne, a 65 year old business man who has presented with chest pain

**History of presenting complaint:** He said the pain started at 9am this morning and has been intermittent. The pain is located in in the centre of his chest and radiates to his jaw. He described this pain as crushing and rated it as 9/10. He has associated shortness of breath, sweating and is feeling nauseous. He has vomited twice since the pain started. The pain is present at rest and is worse on exertion. The pain is relieved by his GTN spray.

**Past medical history:** He has a background of Angina, Type 2 Diabetes and Osteoarthritis.

**Relevant family history:** His father died of a heart attack when he was 54 and his mother also had Type 2 Diabetes

**Relevant social history:** He is a heavy smoker and has smoked 15 cigarettes a day for the past 40 years. He also has a diet high in salt intake.

**Drug history & any allergies:** He currently takes Metformin, Atorvastatin, and Ramipril. He has a penicillin allergy which I will document.

**Ideas concerns and expectations:** His main concern is whether he is having a heart attack and would like the pain to go away.

**Differential diagnosis:** Based on his history and significant cardiovascular risk factors, my top differential is that he is having an acute coronary syndrome. Other differentials that I would like to exclude are a pulmonary embolism and stable angina.

**ROLE:** Second Year Medical Student

**LOCATION:** Medical Admissions Unit

**SCENARIO:** You have been asked to speak to Mr/Mrs Parker who has presented with palpitations. You have 7 minutes to take a history and then you will be asked to present the history to the examiner

# Palpitations history

Follow similar structure and similar questions as chest pain history

## Key Questions to remember:

**HPC:** What do you mean by palpitations? Awareness of heart beat?
When do they start coming on? How often do they come? Did it come on suddenly?
How long do they last for? What were you doing when it started?
Are they regular or irregular? Do you feel like you skip a heartbeat? Can you tap the rhythm?
Is there anything obvious that triggers the palpitations? Is there anything that resolves it?
Is there anything that makes the palpitations better/worse?
Are there any other symptoms? Dizziness? SOB? Chest pain? Leg Swelling? Tremor?
Heat intolerance? Weight loss? Low mood? + SYSTEMS REVIEW

**PMH:** Any history of Diabetes, HTN, Thyroid disease, Irregular heart beat? Mental Health?

**FH:** Any history of Diabetes, HTN, Thyroid disease, irregular heart beat?

**DH:** Caffeine intake? Beta-agonists? Beta blockers? QT prolonging drugs? ALLERGIES?

**SH:** Smoking, Alcohol, Recreational drugs (cocaine)
ASK ABOUT STRESS and any recent emotional/physical trauma

**ICE:** SHOULD BE DYNAMIC

| Arrhythmia e.g. atrial fibrillation | Hyperthyroidism | Anxiety |
|---|---|---|
| PALPITATIONS | PALPITATIONS | PALPITATIONS |
| IRREGULAR RHYTHM WHEN THEY TAP IT | SWEATING/ HEAT INTOLERANCE/ WEIGHT LOSS/ EYE SYMPTOMS | SWEATING/ DIZZINESS/ TIGHTNESS |
| DIZZINESS/SOB/SYNCOPE | HISTORY OF OTHER AUTOIMMUNE DISEASES E.G. TYPE 1 DIAEBTES | HISTORY OF STRESS |
| HISTORY OF CARDIOVASCULAR DISEASE/ THYROID DISEASE? | | HISTORY OF EMOTIONAL/PHYSICAL TRAUMA |

# Performing an ECG

WIPE

CHAPERONE FOR FEMALE PATIENT

EXPOSE (offer shaving/ wiping chest hair)

'Ride your green bike'

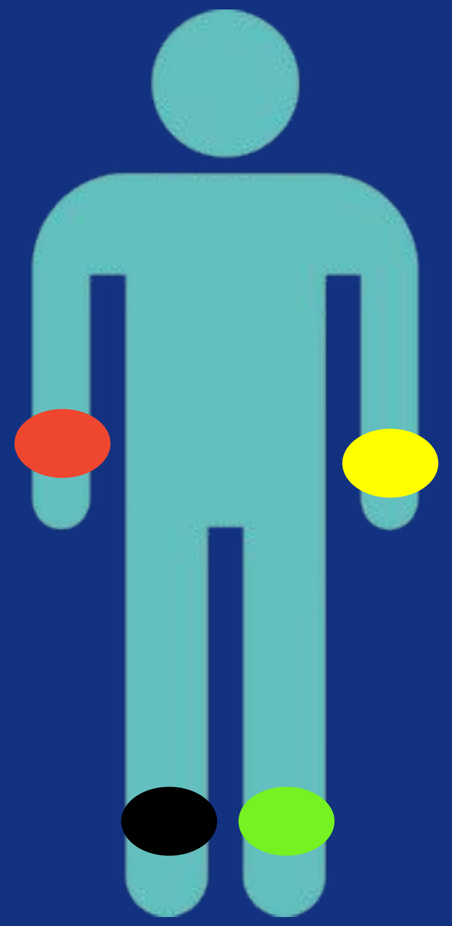

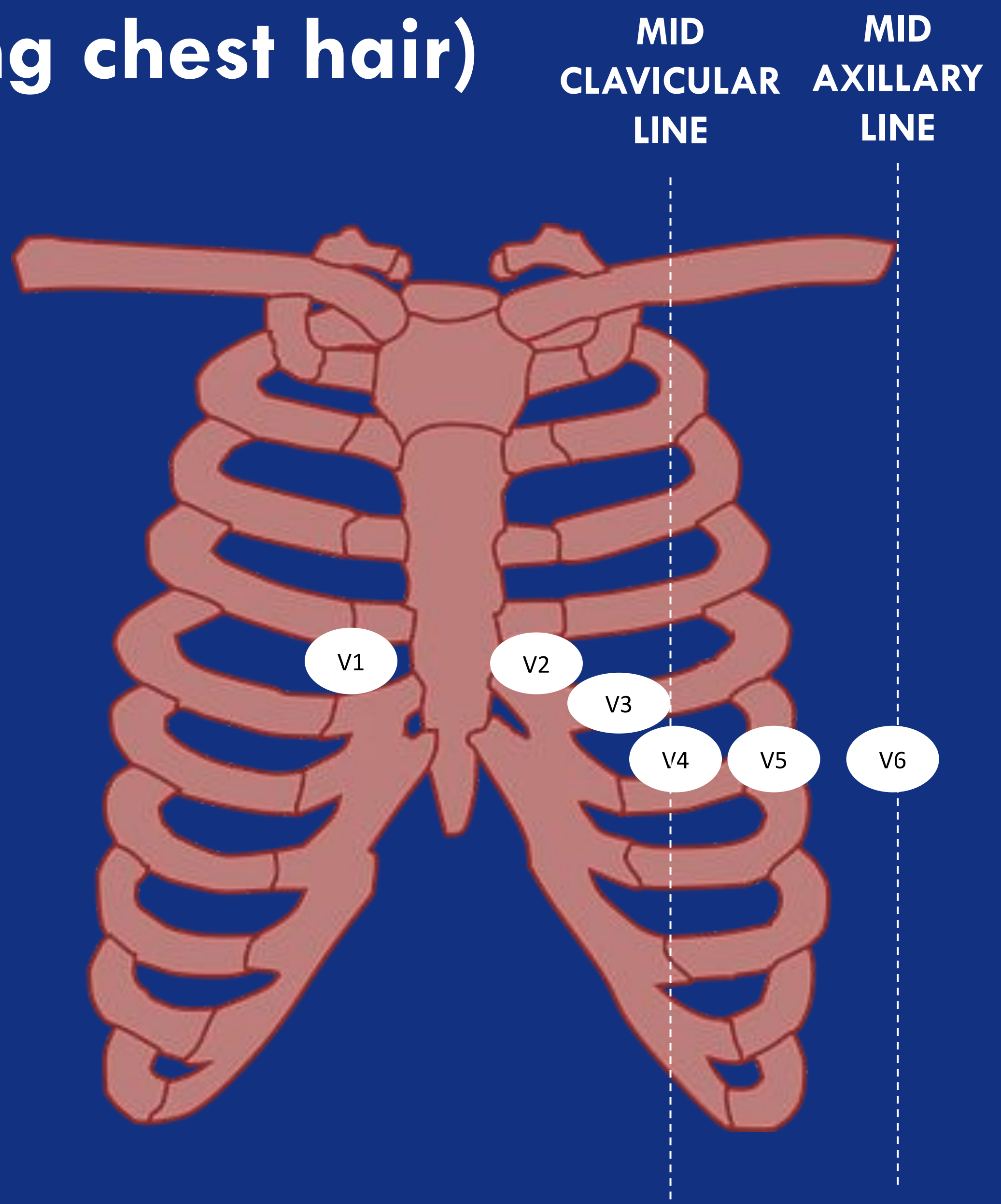


After completing, remove stickers & leads

Thank the patient and label the ECG

# Basic ECG Interpretation

**Rate:** Divide 300 by the number of big squares between the R-R interval
**OR**
Count number of QRS complexes in 30 squares and then multiply by 10

**Rhythm:** Are p waves present? Is each p wave associated with the QRS? **= SINUS RHYTHM**
Regular?, Regularly irregular?, Irregularly irregular

**P waves:** Check if present and if normal morphology

**PR Interval:** Should be 3-5 small squares

**QRS complex:** Should be less than 3 small squares
Narrow or broad?

**ST segment:** Check for elevation and depression. Check for reciprocal changes

**T waves:** Check for inversion or very tall. Inversion is normal in aVR, V1 and III

**QT interval:** Check the QTc time at top of ECG. Usually < 450 ms

**Axis:** Check for left/right axis deviation

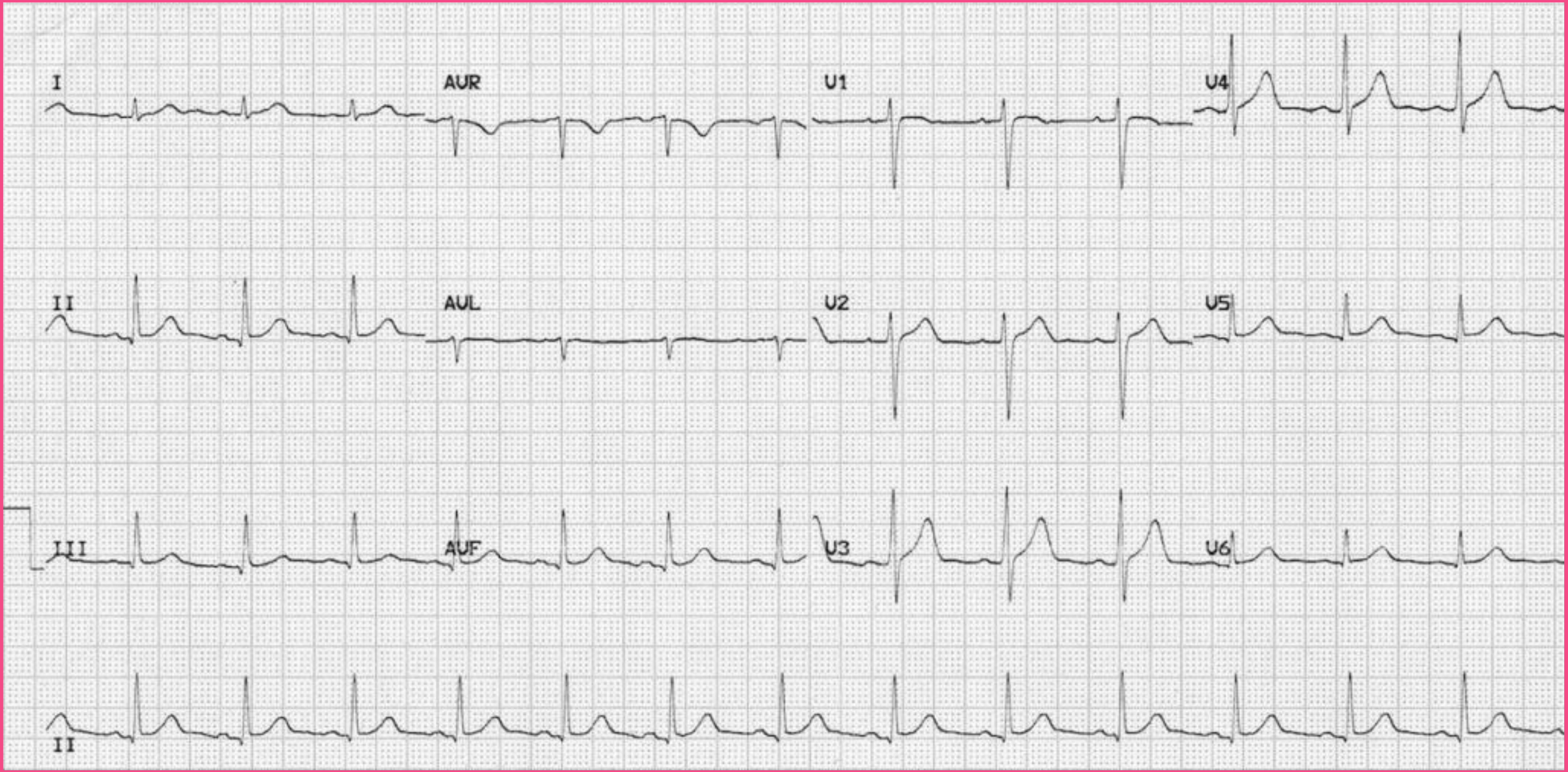


CREDIT - litfl.com

# PRESENTING THE ECG

**Name, DOB, Symptoms:** This is an ECG of Tony Stark, a 48 year man, who has chest pain

**ECG date and time:** The ECG was taken today at 12.43pm

**Type of ECG:** The ECG is calibrated at 25mm/s

**Any obvious abnormalities:** No obvious acute ischaemic changes

**Rate:** The rate is 84 beats per minute

**Rhythm:** The rhythm is regular

**P waves:** There are p waves present and they are associated with each QRS and it appears in sinus rhythm

**PR Interval:** The PR interval is normal

**QRS complex:** The QRS complexes are normal and narrow

**ST segment:** There are no ST segment changes

**T waves:** There is T wave inversion in aVR

**QT interval:** The QTc interval is … and is not prolonged

**Axis:** The axis is normal

**Summarise ECG:** This ECG shows a sinus rhythm

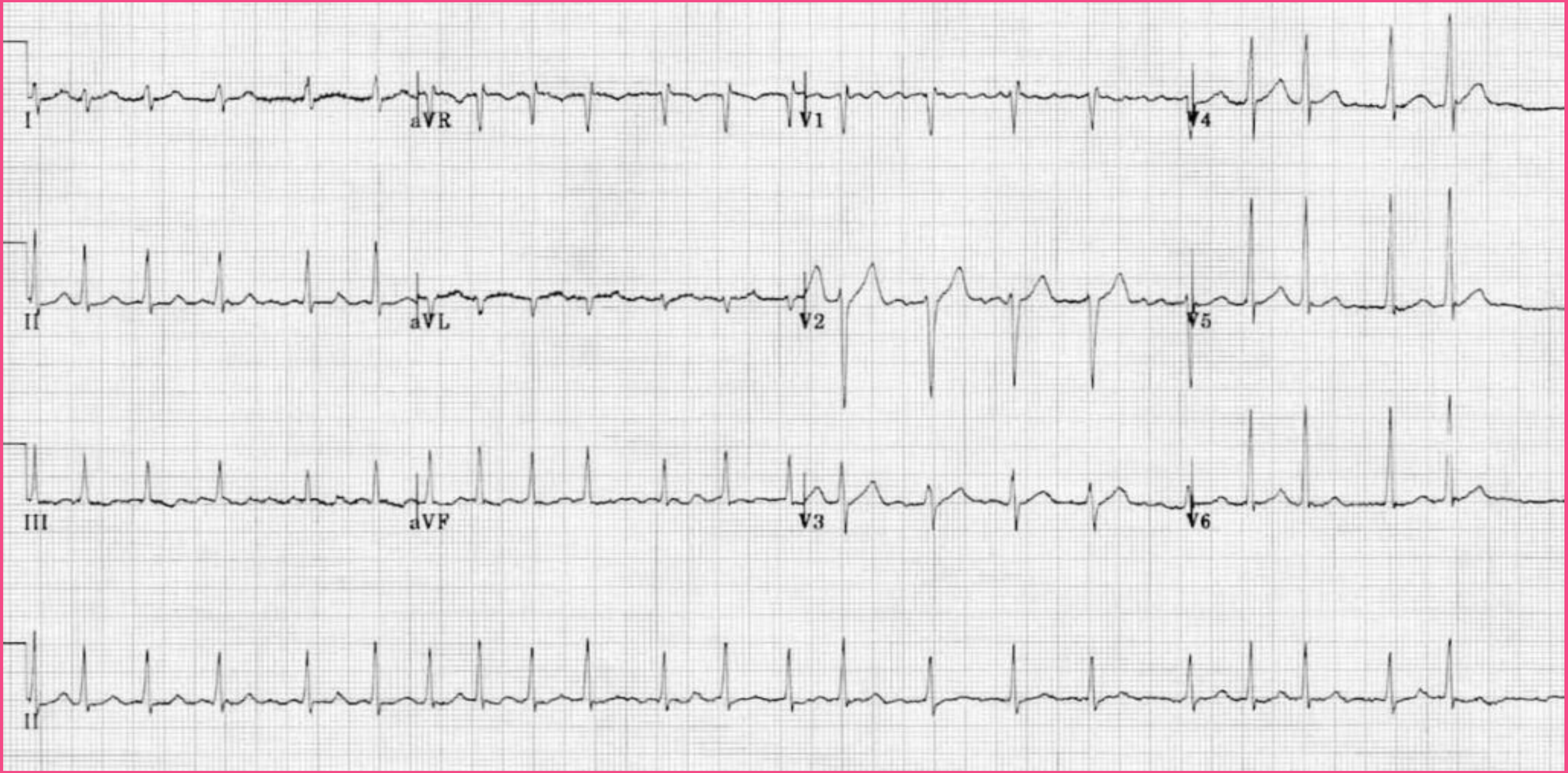


CREDIT - litfl.com

# PRESENTING THE ECG

**Name, DOB, Symptoms:** This is an ECG of Steve Rodgers, a 95 year man, who has palpitations

**ECG date and time:** The ECG was taken today at 4.30pm

**Type of ECG:** The ECG is calibrated at 25mm/s

**Any obvious abnormalities:** No obvious acute ischaemic changes

**Rate:** The rate is 80 beats per minute

**Rhythm:** The rhythm is irregularly irregular

**P waves:** There are no p waves present which means the rhythm is not sinus

**PR Interval:** As there are no p waves I cannot determine PR interval

**QRS complex:** The QRS complexes are normal and narrow

**ST segment:** There is no ST segment changes

**T waves:** There is T wave inversion in aVR

**QT interval:** The QTc interval is … and is not prolonged

**Axis:** The axis is normal

**Summarise ECG:** This ECG shows that the patient has a tachycardia with an irregularly irregular rhythm and no visible p waves. This is indicative of atrial fibrillation with rapid ventricular response

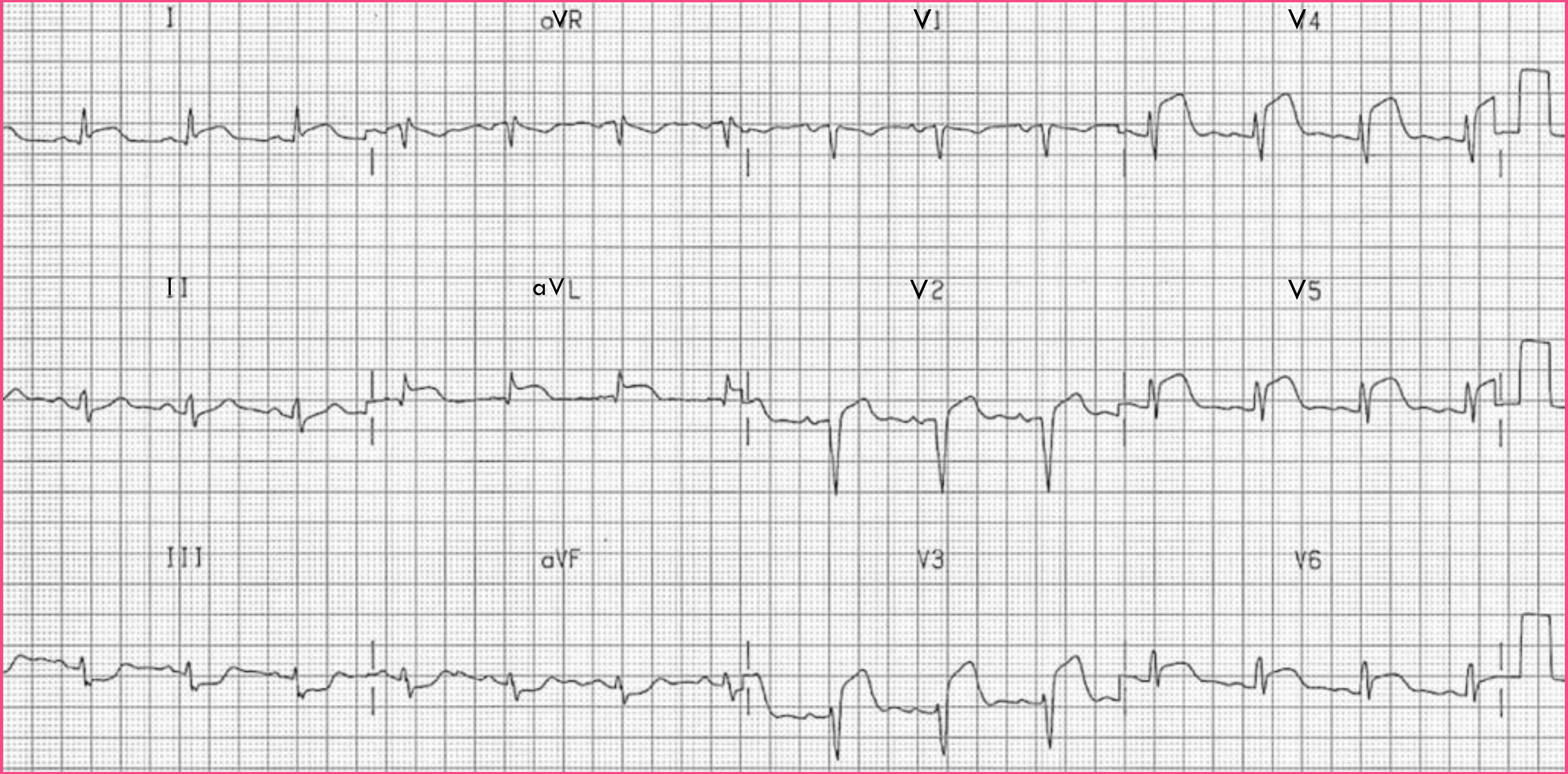


CREDIT - litfl.com

**ANTEROSEPTAL – LAD**

**LATERAL – Left Circumflex**

**Inferior – Right coronary artery**

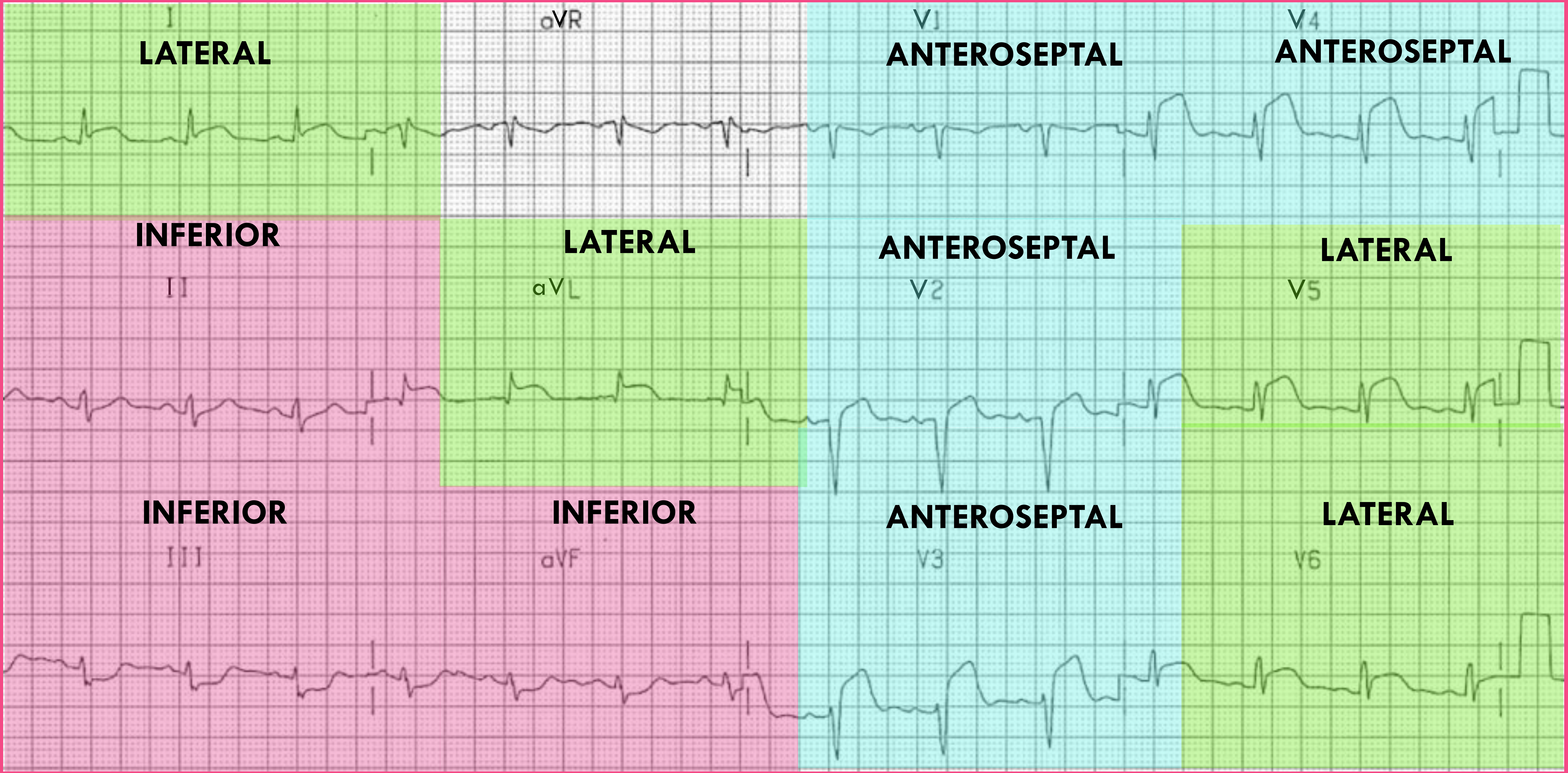


**Posterior – Left or Right coronary artery**

CREDIT - litfl.com

# PRESENTING THE ECG

**Name, DOB, Symptoms:** This is an ECG of Thor, a 1000 year man, who has chest pain

**ECG date and time:** The ECG was taken today at 10.33pm

**Type of ECG:** The ECG is calibrated at 25mm/s

**Any obvious abnormalities:** There are clear acute ischaemic changes

**Rate:** The rate is 84 beats per minute

**Rhythm:** The rhythm is regular

**P waves:** There are p waves present and they are associated with each QRS and it appears in sinus rhythm

**PR Interval:** The PR interval is normal

**QRS complex:** The QRS complexes are narrow

**ST segment:** There is clear ST elevation in V2-V6, Lead I and aVR
There is also reciprocal ST depression in Lead III and aVF

**T waves:** There is T wave inversion in aVR and VI

**QT interval:** The QTc interval is … and is not prolonged

**Axis:** The axis is normal

**Summarise ECG:** This ECG shows ST elevation in the anterolateral leads with reciprocal ST depression in the inferior leads. The left coronary artery is likely occluded.

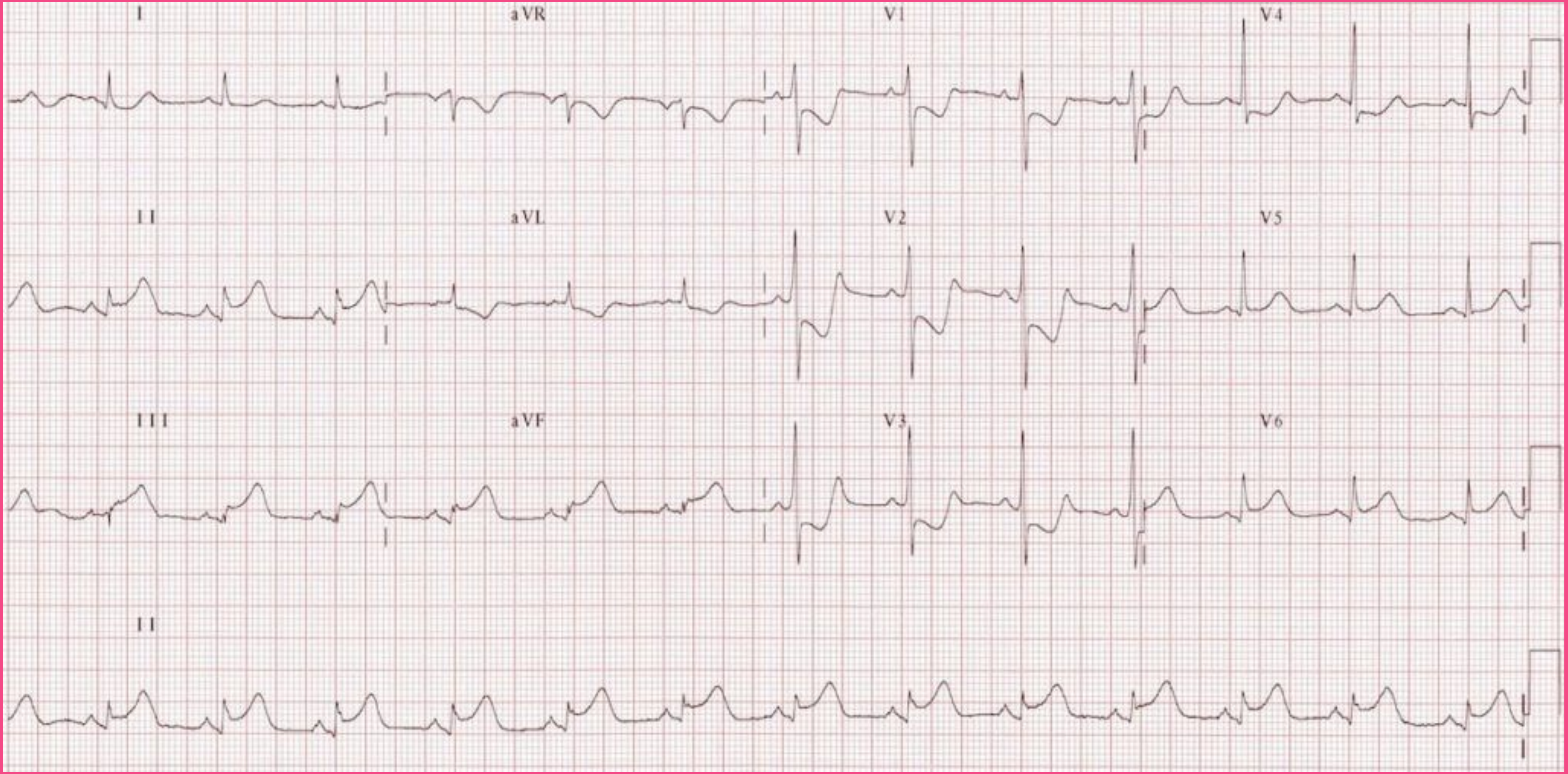


CREDIT - litfl.com

# Reciprocal changes

| STEMI Location | Reciprocal ST depression |
|---|---|
| Anterolateral | Inferior leads |
| Lateral | Inferior leads |
| Inferior | Lateral leads |
| Posterior | Anterior leads |

# PRESENTING THE ECG

**Name, DOB, Symptoms:** This is an ECG of Natasha Romanoff, a 45 year woman with chest pain

**ECG date and time:** The ECG was taken today at 5.30pm

**Type of ECG:** The ECG is calibrated at 25mm/s

**Any obvious abnormalities:** There are clear acute ischaemic changes

**Rate:** The rate is 75 beats per minute

**Rhythm:** The rhythm is regular

**P waves:** There are p waves present and they are associated with each QRS and it appears in sinus rhythm

**PR Interval:** The PR interval is normal

**QRS complex:** The QRS complexes are narrow

**ST segment:** There is clear ST elevation in Lead II, III and aVF
There is also ST depression in V1 – V4

**T waves:** There is T wave inversion in aVR and aVL.

**QT interval:** The QTc interval is … and is not prolonged

**Axis:** The axis is normal

**Summarise ECG:** This ECG shows ST elevation in the inferior leads and ST depression in the anterior leads. I would like to place V7,V8 and V9 leads to also check for Posterior STEMI.

# Cardiac Blood Test

| | |
|---|---|
| Haemoglobin | 15 (13.0 - 17.0 g/dL) |
| White Blood Cells | 90 (3.0 - 10.0 x $10^9$/L) |
| Mean Corpuscular volume | 90 (80 - 96 fL) |
| CRP | 600 (< 5 mg/L) |
| Troponin | 1000 (25 - 200 U/L) |
| Creatinine Kinase | 100 (<0.01 µg/L) |
| Urea | 5 (2.5 - 7.8 mmol/L) |
| Sodium | 143 (135 - 146 mmol/L) |
| Potassium | 4.1 (3.5 - 5.5 mmol/L) |

# PRESENTING THE BLOOD TEST

This is a blood test of Barry Allen, a 24 year old man who has chest pain. The key positive findings from this blood test is raised white blood cells with significantly raised CRP, Troponin and Creatine Kinase levels. The raised  Troponin and Creatine Kinase indicates cardiac damage likely due to an acute coronary syndrome. The raised CRP also indicates acute inflammation.

In summary, this blood test shows that the patient likely has a STEMI or an NSTEMI depending on the ECG findings. I would like to check the patient's most recent ECG to confirm the diagnosis.

**ROLE:** Second Year Medical Student

**LOCATION:** Resus

**SCENARIO:** You have been asked to speak to Mr/Mrs Smith who has presented with chest pain. You have 7 minutes to perform a cardiovascular examination and then you will be asked to present your findings to the examiner

# TIPS FOR ALL PHYSICAL EXAMINATIONS

For general inspection, LOOK at the patient and around the bed for a good few moments

When talking in between the examination, say ‘’there is no evidence of….’’ rather than ‘’I am looking for …’

Always perform WIPEE

Examine from the patient’s right side

BE SYSTEMATIC and try to look slick

PUT ON A SHOW!!!

NEVER PALPATE CAROTIDS BILATERALLY AT SAME TIME

# The Cardiovascular examination

## WIPEE

''Hello, my name is …. and I am a second year medical student. Can I confirm your name and age please.

Today, I've been asked to perform an examination of your heart. What that will involve is me having a general look of you, examining your hands, face and back and also have a listen to your chest. Does that sound okay? Do I have your consent?

Just to let you know, I will also be talking out load to the examiner while I perform the examination to let him/her know what I am looking for.

Are you in any pain at the moment? Where is the pain?

The examination should not be painful but if you feel any discomfort, please let me know.

# General Inspection

Comfortable at rest?

Any peripheral stigmata of cardiovascular disease such as medications like GTN spray, oxygen or mobility aids

# Hands

Check capillary refill time

Any digital clubbing (ABC), tendon xanthomata, Osler nodes, Janeway lesions, Splinter haemorrhages and peripheral cyanosis?

Comment on rate, rhythm and character of pulse

Ask if patient has shoulder pain!!!!!

Perform a collapsing pulse

Offer taking blood pressure both sides as well standing and sitting blood pressure

# Neck & Face

Make sure patient is sitting at 45 degrees

Check if JVP is elevated

Listen before feeling carotids.

Look at eyes for pallor, corneal arcus, xanthelasma

Say what you would examine inside mouth  do not take off patient's mask

# Chest

Spend some time looking at the chest and axilla for any scars (Check legs for CABG scar)

Palpate for thrills over auscultation areas

Palpate for heaves next to sternum

Palpate apex beat and check if in correct position

Auscultate all four areas whilst palpating the carotid

Roll patient onto left side and auscultate axilla

Sit patient forward and auscultate aortic area

# Back & Legs

Listen to lungs both sides

Check sacral oedema

Check ankle oedema (do not press too hard!)

**Thank patient & restore clothing!**

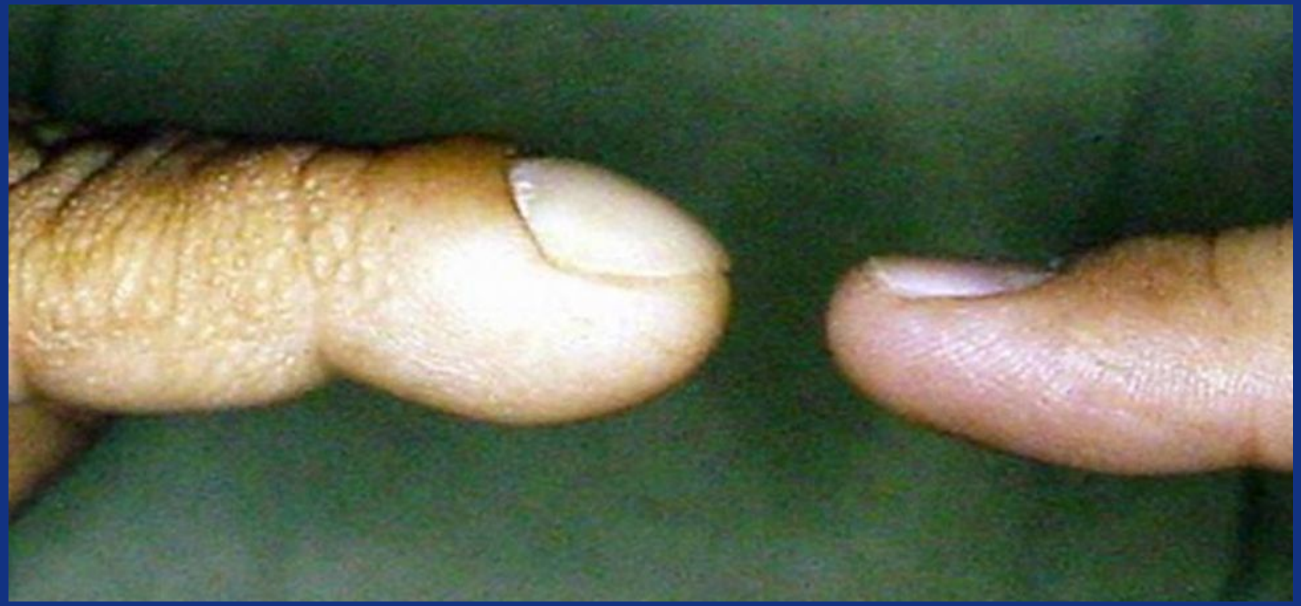
Finger Clubbing

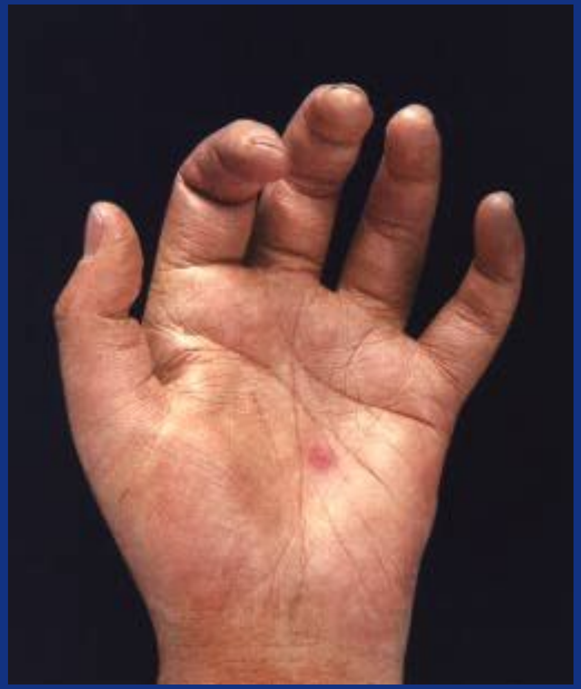
Janeway lesions

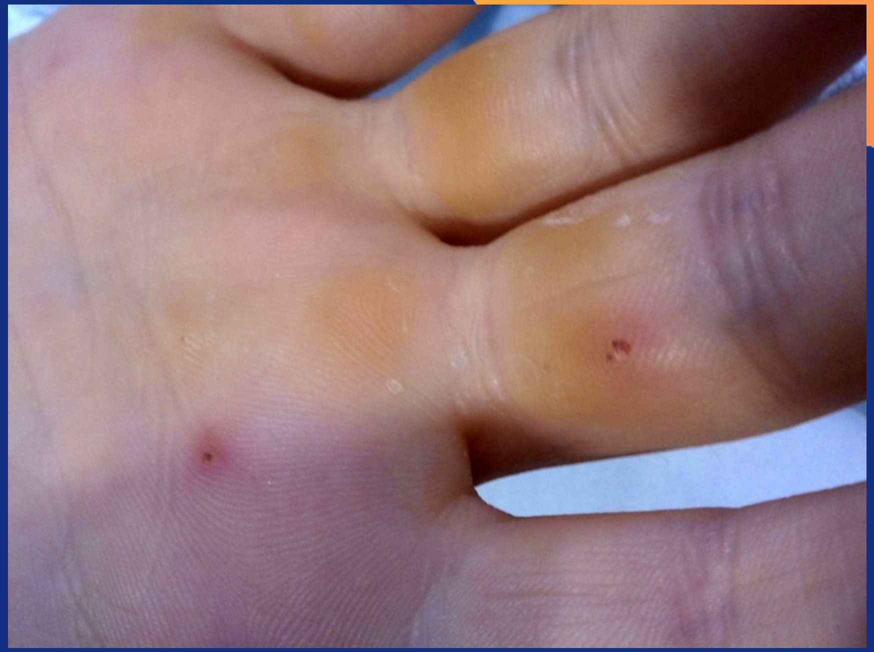
Osler’s nodes

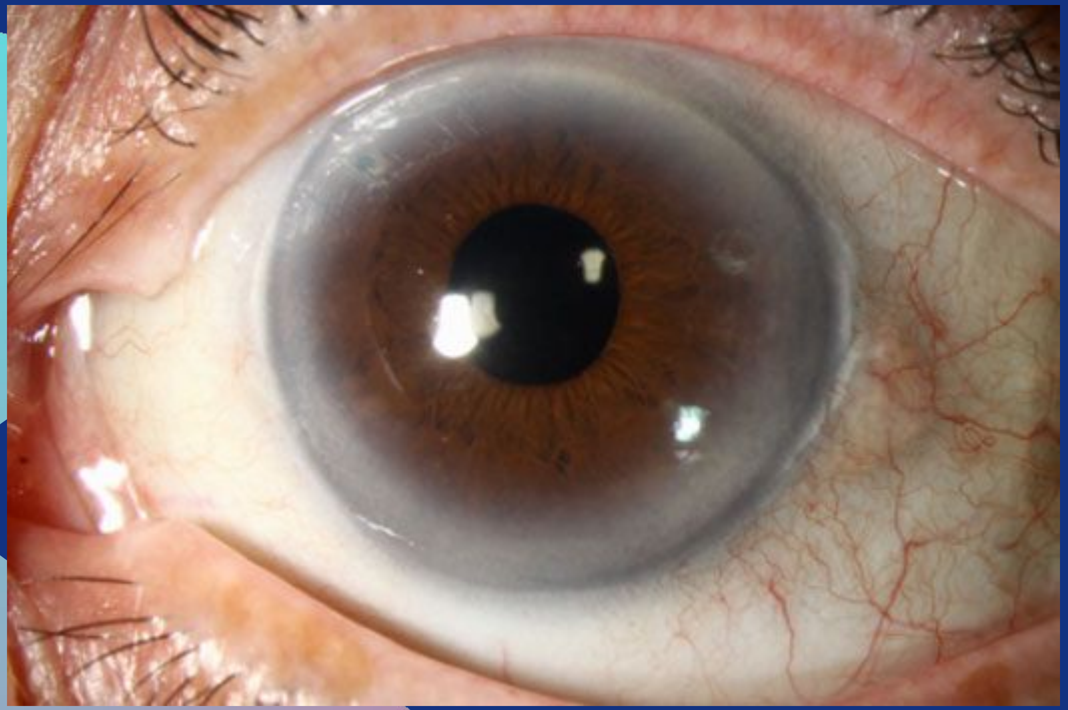
Corneal arcus

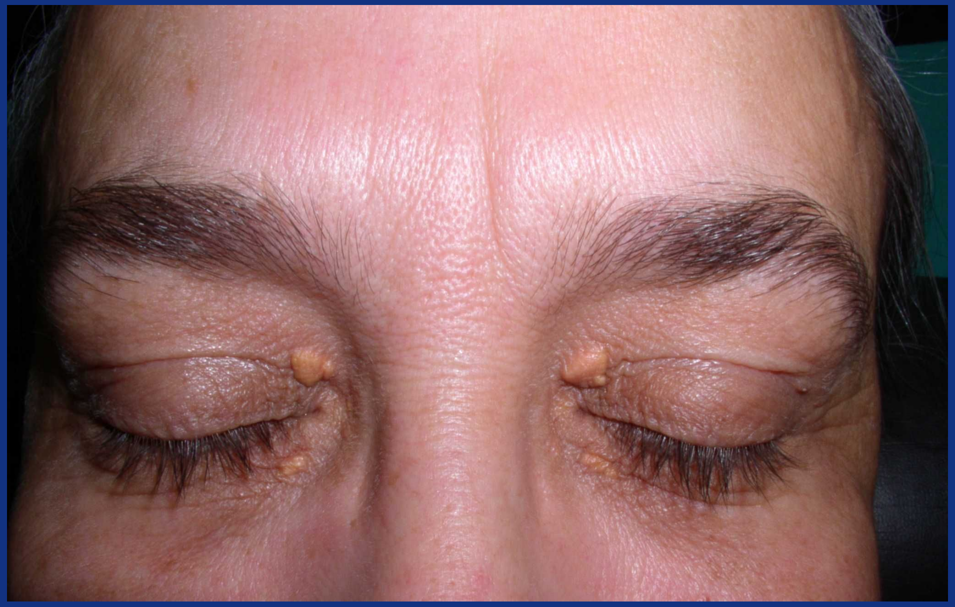
Xanthelasma

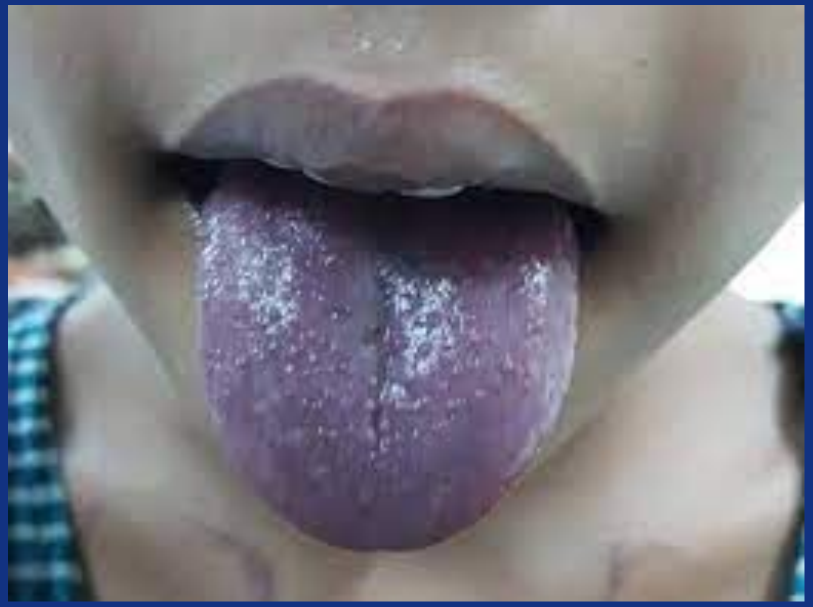
Central cyanosis

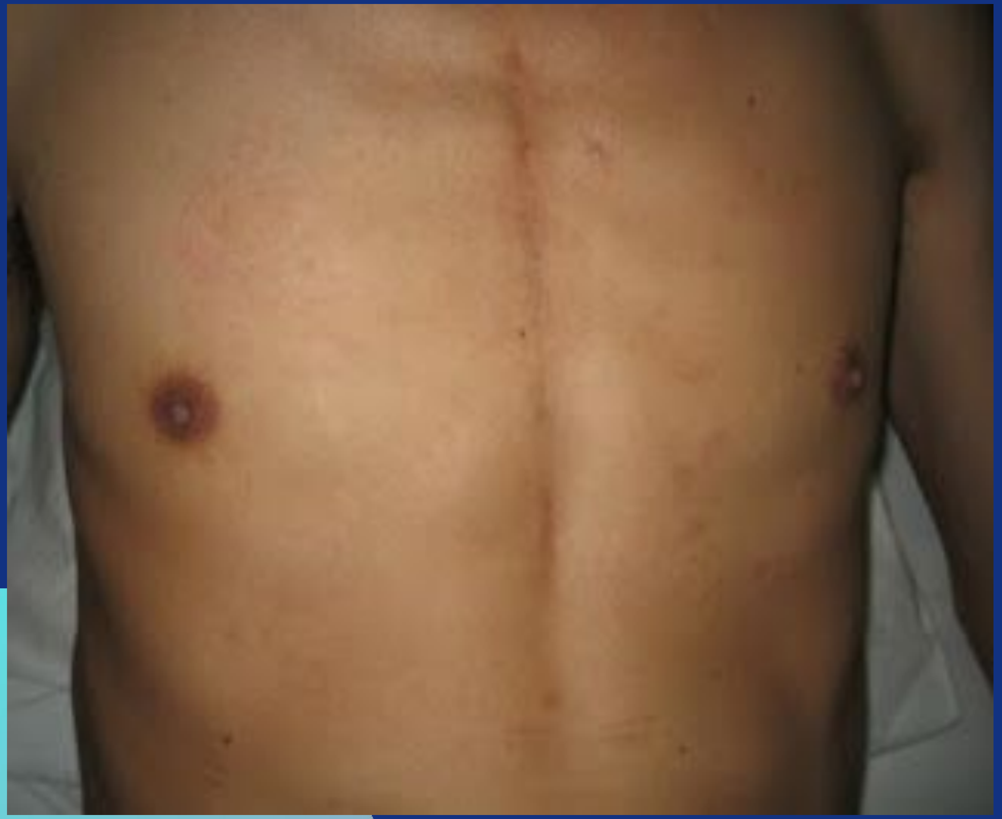


Midline Sternotomy

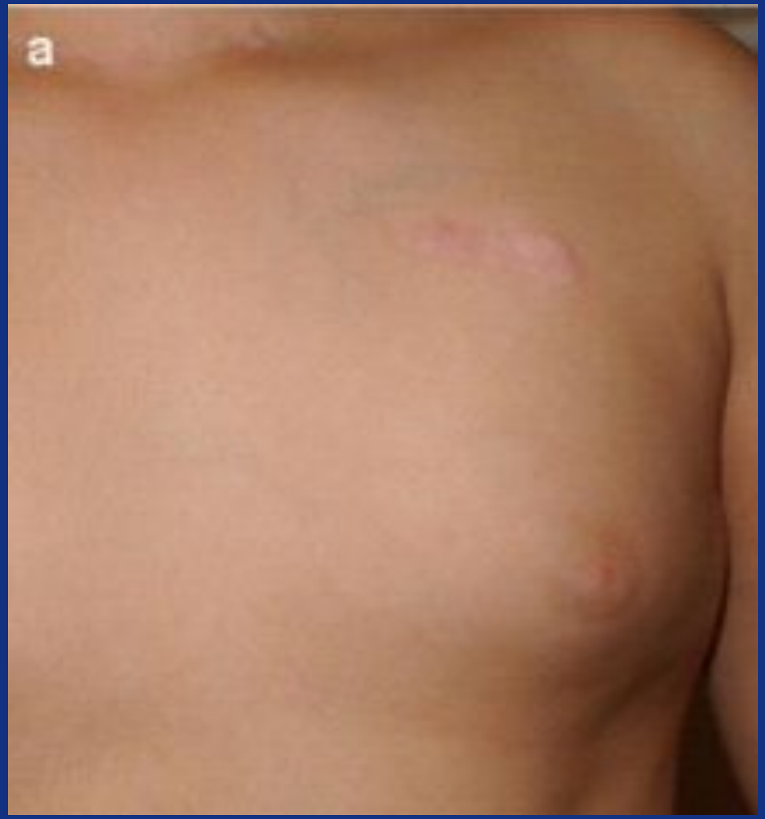


Infraclavicular

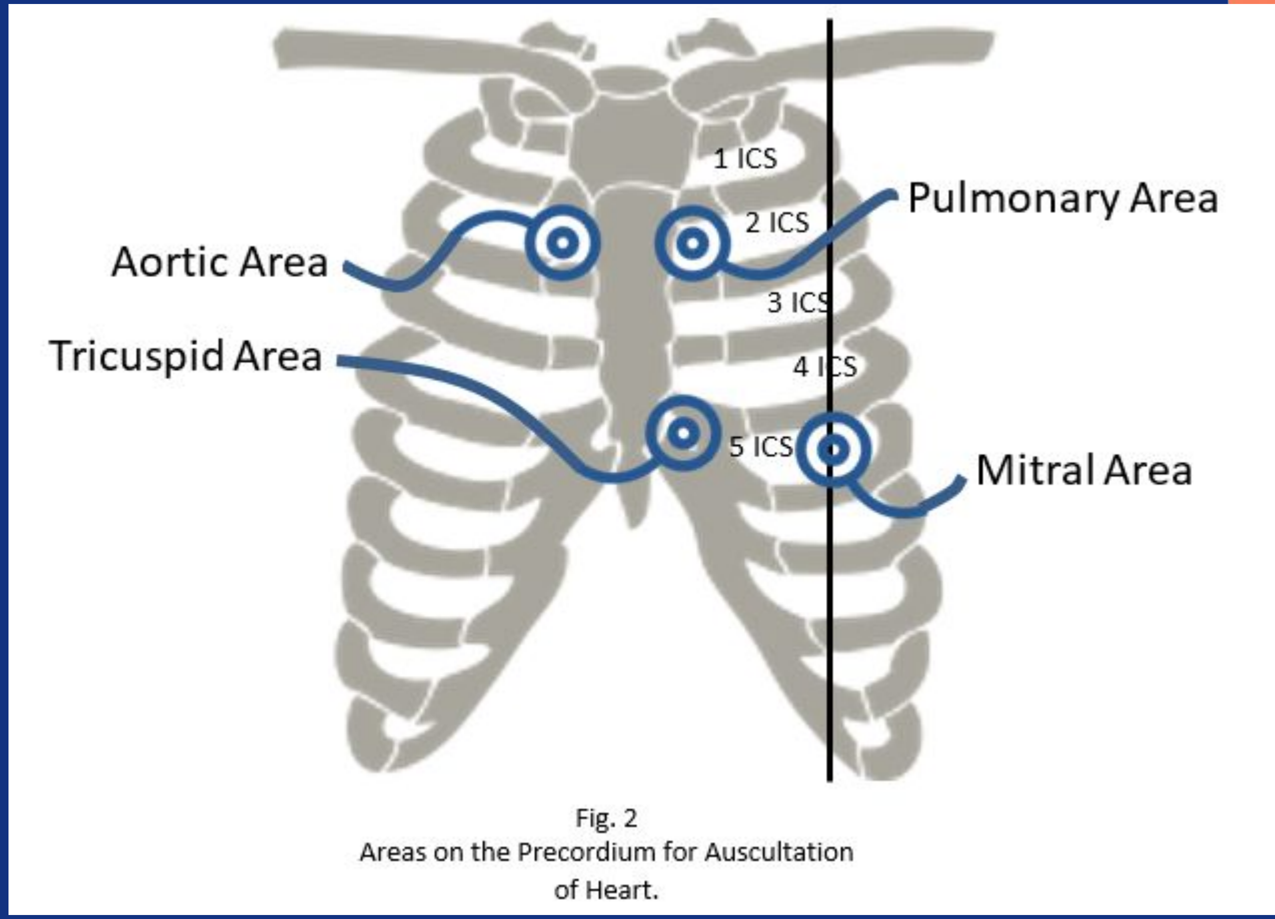


Auscultatory sites

# PRESENTING THE CARDIO EXAM

I performed a cardiovascular examination of Bruce Banner, a 34 year old man.
On general inspection, he was comfortable sitting upright on the bed and seemed alert and orientated.
Around the bed, there no signs of any peripheral stigmata of cardiovascular disease
On examination of the hands, he had a normal capillary refill time and no evidence of clubbing, cyanosis, tendon xanthomata and signs of infective endocarditis
His JVP was not elevated and there was no evidence of cardiovascular disease on examination of his face
On closer inspection of his chest, there were no scars to suggest previous surgeries and there were no chest wall deformities
There were no palpable thrills or heaves and the apex beat was not displaced
On auscultation of the chest, both heart sounds were present and there no added sounds or murmurs
On auscultation of the lung bases, there is no evidence of pulmonary oedema and finally there was no evidence of peripheral oedema
In summary, this was a normal cardiovascular examination.

To complete my examination, I would like to take a formal history and a full set of observations including an ECG. I would like to also perform an examination of the respiratory system as well as the peripheral arterial system. I would like to perform fundoscopy to check for signs of infective endocarditis or hypertension. Finally, I would like to perform urinalysis to check for proteinuria and haematuria.

THANK YOU FOR LISTENING!

FILL IN THE POST-SESSION QUIZ & FEEDBACK FORM FOR TODAY'S SLIDES

GET IN CONTACT WITH YOUR TUTOR AND PRACTISE!
FILL IN THE TUTOR FEEDBACK FORMS AND GET THIS WEEK's SCENARIO MARK SHEET AND EXAMINATION CHEAT SHEET

SEE YOU NEXT WEEK FOR RESPIRATORY!
FOLLOW US ON FACEBOOK FOR ALL UPDATES
EMAIL ENQUIRIES: isceazy1to2@gmail.com

# CARDIOLOGY

Written By Movin Peramuna Gamage

# STUDENT INSTRUCTIONS

| | |
|---|---|
| Role | 2nd year medical student |
| Setting | A&E |
| Patient | Mr Calvin, a 61 y/o male presents with acute chest pain. |
| Student task | Take a concise history from the patient regarding his presenting symptoms.<br><br>At 7 minutes, the examiner will stop you, ask you to summarise your findings and present a differential diagnosis.<br><br>Then you will carry out an examination and interpret some clinical data. Finally, you will discuss management (formative). |

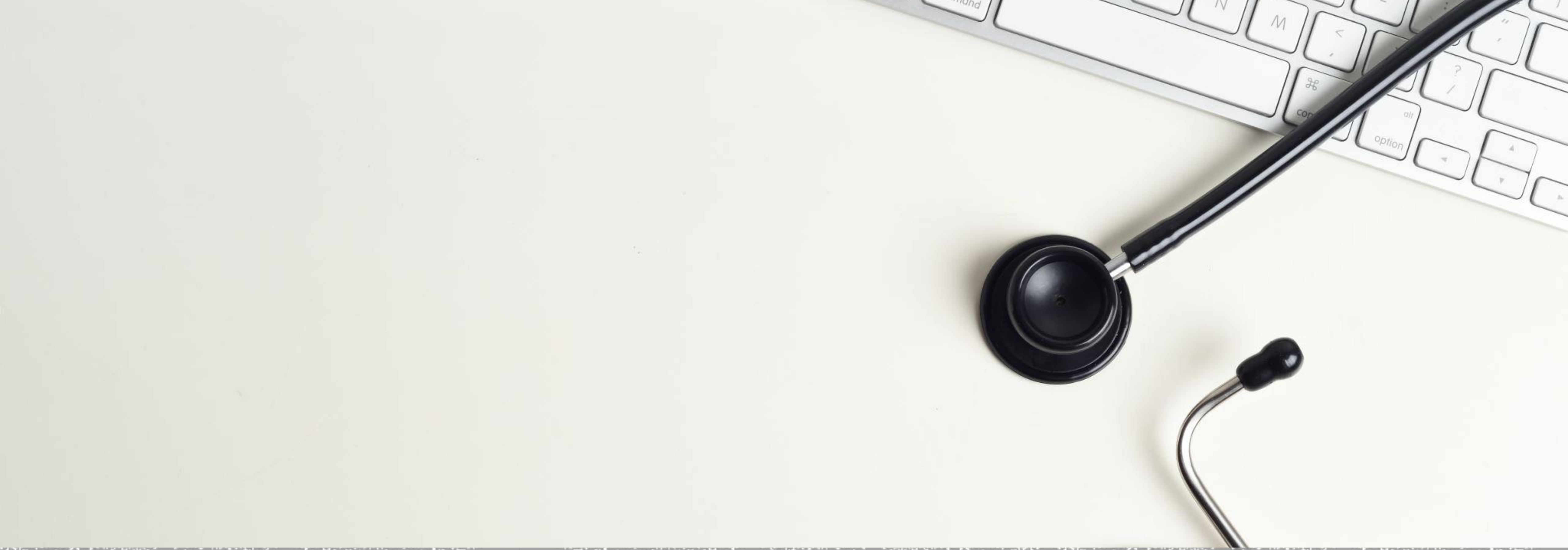

# PATIENT HISTORY - TUTOR

| | |
|---|---|
| PC | Mr John Calvin, a 61 y/o male presents with acute chest pain. (DOB: 02/05/1960) |
| HPC | • Pain (central location, came on 2 hours ago while reading a book, tight sensation/"like an elephant sitting on your chest", radiates to jaw, intermittent dizziness and vomiting, nothing makes pain better or worse, 6/10 on severity scale)<br>• Has been recently feeling short of breath<br>• No prior chest pain |
| PMHx | • Hypertension, Familial hypercholesterolaemia, Type 2 diabetes mellitus<br>• No surgical Hx |
| FHx | His father passed away due to a MI at age 58. Father also had high cholesterol levels. Mother passed away due to pancreatic cancer. |
| SHx | • Ex-smoker (used to smoke 30 a day until the age of 54, started smoking at age of 14)<br>• Consumes alcohol recreationally (occasional beer over a rugby game)<br>• Lives with wife but is independent and mobile<br>• Retired technician<br>• Does not exercise but can usually manage going to the shops |
| Drug Hx | • Ramipril, amlodipine, indapamide, atorvastatin, metformin<br>• No known drug allergies |
| ICE | • Idea – possibly severe reflux or heart attack<br>• Concerns – worried about pain<br>• Expectations – pain relief, want to know cause of presentation |

| | |
|---|---|
| Differential diagnosis | ACS, Pulmonary embolism and Aortic dissection |
| Final diagnosis | ACS |

# MARK SCHEME – HISTORY

| | | |
|---|---|---|
| **INTRODUCTION** | | |
| 1 | Introduces themselves | |
| 2 | Confirms patient details | |
| 3 | Establishes presenting complaint using open questioning | |
| **HISTORY OF PRESENTING COMPLAINT** | | |
| 4 | Onset / Duration | |
| 5 | Severity | |
| 6 | Intermittent / Continuous | |
| 7 | Exacerbating / Relieving factors | |
| 8 | Associated symptoms | |
| 9 | Ideas / Concerns / Expectations | |
| **KEY SYMPTOMS** | | |
| 10 | Chest pain | |
| 11 | Dyspnoea | |
| 12 | Palpitations | |
| 13 | Syncope | |
| 14 | Oedema | |
| **CARDIOVASCULAR RISK FACTORS** | | |
| 15 | Hypertension | |
| 16 | Hyperlipidaemia | |
| 17 | Diabetes | |
| 18 | Smoking | |
| 19 | Family history of cardiovascular disease | |
| **PAST MEDICAL HISTORY** | | |
| 20 | Previous cardiovascular disease | |
| 21 | Other medical conditions | |
| 22 | Surgical history | |
| **DRUG HISTORY** | | |
| 23 | Prescribed medications | |
| 24 | Over the counter medication | |
| 25 | ALLERGIES | |

| | | |
|---|---|---|
| **FAMILY HISTORY** | | |
| 26 | Cardiovascular disease (*including age of onset*) | |
| **SOCIAL HISTORY** | | |
| 27 | Smoking history / Alcohol intake / Recreational drug use | |
| 28 | Home situation / Level of functional independence | |
| 29 | Occupation | |
| **SYSTEMIC ENQUIRY** | | |
| 30 | Screens for symptoms in other body systems | |
| **CLOSING THE CONSULTATION** | | |
| 31 | Thanks patient | |
| 32 | Summarises salient points of the history | |
| **KEY COMMUNICATION SKILLS** | | |
| 33 | Active listening | |
| 34 | Summarising | |
| 35 | Signposting | |

# CARDIOVASCULAR EXAMINATION – STUDENT

- Student instructions: Briefly go over the procedure for a normal cardiovascular examination

# MARK SCHEME – EXAMINATION

| | | |
|---|---|---|
| 1 | Washes hands | |
| 2 | Introduces self & explains examination | |
| 3 | Gains consent | |
| 4 | Positions and exposes patient appropriately | |
| 5 | Performs general inspection | |
| 6 | Inspects & assesses hands - *clubbing / temperature / CRT* | |
| 7 | Assesses radial pulse - *rate / rhythm / radial-radial delay / collapsing pulse* | |
| 8 | Assesses brachial pulse & offers to record blood pressure | |
| 9 | Assesses carotid pulse appropriately | |
| 10 | Observes JVP & checks for hepatojugular reflux | |
| 11 | Inspects eyes - *Xanthelasma / Corneal arcus / Conjunctival pallor* | |
| 12 | Inspects mouth for central cyanosis | |
| 13 | Inspects precordium | |
| 14 | Palpates for heaves, thrills and apex beat | |
| 15 | Auscultates all heart valves appropriately whilst feeling carotid pulse | |
| 16 | Auscultates carotids, left sternal edge & axilla for radiation of murmurs | |
| 17 | Performs accentuation manoeuvres | |
| 18 | Auscultates lung bases, inspects for sacral oedema & assess peripheral oedema | |
| 19 | Thanks patient | |
| 20 | Washes hands | |

**EXAMINER**

"*Summarise your findings, suggest further investigations and offer a differential diagnosis*"

| | | |
|---|---|---|
| 20 | Accurately summarises salient findings | |
| 21 | Suggests appropriate further investigations | |
| 22 | Suggests appropriate differential diagnosis | |

# DATA INTERPRETATION - STUDENT

- Student instructions: Please interpret the following ECG

Name: John Calvin
DOB: 02/05/1960
Date taken: 05/03/2021
Time taken: 12.05 pm

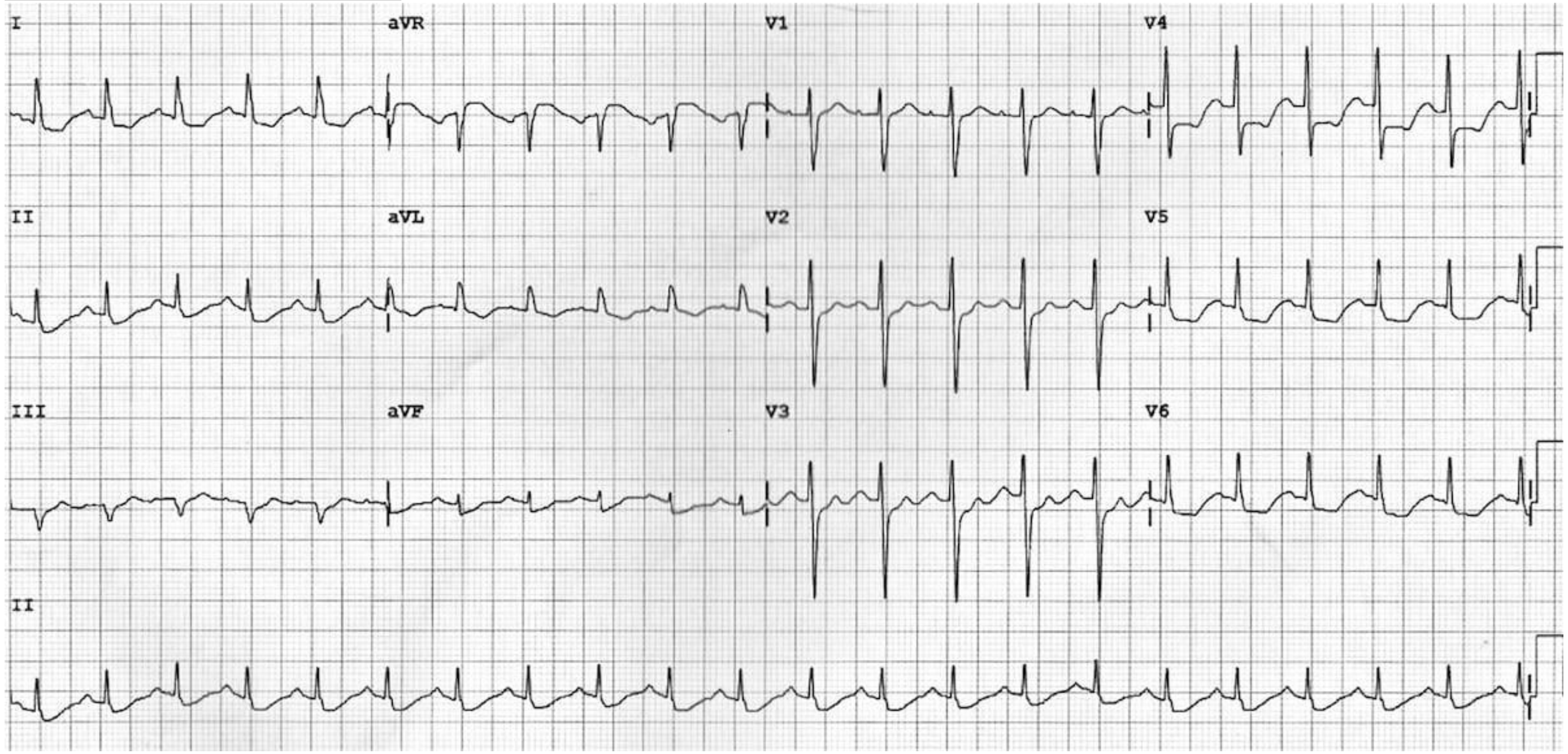

# DATA INTERPRETATION- MARK SCHEME

- Confirm patient details – Name, DOB, date and time of ECG taken
- Determine rate and rhythm using rhythm strip – 130 bpm (tachycardia), regular rhythm
- Check cardiac axis – Normal axis (I and aVF)
- ST segment depression in lead I, II, aVF and V4-V6 (widespread ischaemia)

- ECG shows NSTEMI (troponin is elevated)

# TESTS AND MANAGEMENT - ACS

| | |
|---|---|
| **Blood Tests** | FBC, U&Es, CRP, Troponin T + I, creatine kinase, blood glucose, lipid profile |
| **Imaging** | CXR (signs of HF), coronary angiography |
| **Management: immediate** | • MONA (Morphine, O2 if sats < 94%, GTN, aspirin), antiemetics<br>• Add ticagrelor (2nd antiplatelet) in ACS with ST changes and elevated troponin<br>• PCI, fibrinolysis |
| **Management: long-term** | • Conservative: healthy diet, weight loss, smoking cessation<br>• Medical: Antiplatelet (e.g. aspirin, add clopidogrel if need be), statins, ACE-i, beta-blocker |
